# Small-molecule eRF3a degraders rescue *CFTR* nonsense mutations by promoting premature termination codon readthrough

**DOI:** 10.1172/JCI154571

**Published:** 2022-09-15

**Authors:** Rhianna E. Lee, Catherine A. Lewis, Lihua He, Emily C. Bulik-Sullivan, Samuel C. Gallant, Teresa M. Mascenik, Hong Dang, Deborah M. Cholon, Martina Gentzsch, Lisa C. Morton, John T. Minges, Jonathan W. Theile, Neil A. Castle, Michael R. Knowles, Adam J. Kimple, Scott H. Randell

**Affiliations:** 1 Marsico Lung Institute and Cystic Fibrosis Research Center,; 2 Department of Cell Biology and Physiology,; 3 Department of Microbiology and Immunology, and; 4 Department of Pediatrics, University of North Carolina at Chapel Hill, Chapel Hill, North Carolina, USA.; 5 Icagen LLC, Durham, North Carolina, USA.; 6 Department of Otolaryngology/Head and Neck Surgery, University of North Carolina at Chapel Hill, Chapel Hill, North Carolina, USA.

**Keywords:** Pulmonology, Therapeutics, Genetic diseases, Ion channels, Translation

## Abstract

The vast majority of people with cystic fibrosis (CF) are now eligible for CF transmembrane regulator (CFTR) modulator therapy. The remaining individuals with CF harbor premature termination codons (PTCs) or rare *CFTR* variants with limited treatment options. Although the clinical modulator response can be reliably predicted using primary airway epithelial cells, primary cells carrying rare *CFTR* variants are scarce. To overcome this obstacle, cell lines can be created by overexpression of mouse Bmi-1 and human TERT (hTERT). Using this approach, we developed 2 non-CF and 6 CF airway epithelial cell lines, 3 of which were homozygous for the *W1282X* PTC variant. The Bmi-1/hTERT cell lines recapitulated primary cell morphology and ion transport function. The 2 *F508del-CFTR* cell lines responded robustly to CFTR modulators, which was mirrored in the parent primary cells and in the cell donors’ clinical response. Cereblon E3 ligase modulators targeting eukaryotic release factor 3a (eRF3a) rescued *W1282X-CFTR* function to approximately 20% of WT levels and, when paired with G418, rescued *G542X-CFTR* function to approximately 50% of WT levels. Intriguingly, eRF3a degraders also diminished epithelial sodium channel (ENaC) function. These studies demonstrate that Bmi-1/hTERT cell lines faithfully mirrored primary cell responses to CFTR modulators and illustrate a therapeutic approach to rescue *CFTR* nonsense mutations.

## Graphical abstract

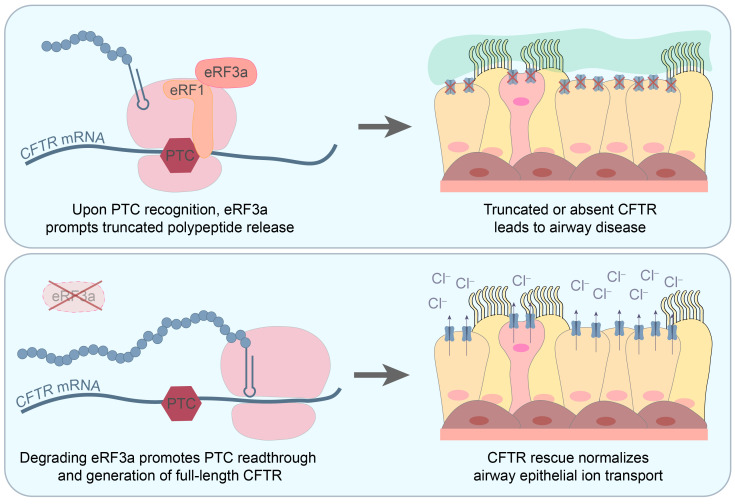

## Introduction

Cystic fibrosis (CF) is a life-limiting genetic disease affecting approximately 70,000 people worldwide (1). Severe pathology develops in the lungs, where absent or dysfunctional CF transmembrane regulator (CFTR) protein leads to the accumulation of thick airway mucus, impaired mucus transport, chronic infection and inflammation, and, eventually, bronchiectasis (2). Historically, treatments have been limited to symptom management. However, the 2012 US FDA approval of the first small-molecule CFTR modulator ivacaftor ushered in a new era of CF precision medicine (3). In contrast to previous treatment approaches, CFTR modulators treat the underlying cause of disease by directly acting on the CFTR protein to correct folding, trafficking, function, or stability. With additional CFTR modulator approvals (4–6) culminating in the 2019 approval of a triple-combination therapy, elexacaftor, tezacaftor, and ivacaftor (trade name Trikafta) (7), as many as 90% of individuals with CF are now eligible for an FDA-approved modulator therapy.

However, developing therapies for the remaining individuals with CF has proven challenging. This is in part because this patient group harbors a wide range of rare *CFTR* variants. Indeed, more than 1200 *CFTR* variants are carried by 5 or fewer individuals worldwide (8, 9). For these individuals, a well-powered clinical trial is not possible. Thus, to extend life-changing treatment to all people with CF, the approach for evaluating candidate therapies must evolve.

The FDA set the precedent for such a change in 2017, when they expanded the use of ivacaftor to patient populations harboring 1 of 23 relatively rare *CFTR* variants (10). Although drug label expansions are common, this instance was particularly groundbreaking because the FDA based their decision purely on in vitro data (11) rather than a clinical trial. This new paradigm relies on the fidelity of in vitro systems to accurately predict clinical responses.

Primary CF human bronchial epithelial cells (HBECs) obtained at the time of lung transplantation have served as the gold standard to assess CFTR rescue in vitro (12–14). However, the supply of CF explant lungs is limited, particularly for rare *CFTR* variants. HBECs can also be obtained by bronchial brushing, but the procedure is invasive and yields low cell numbers (15). A readily available alternative to HBECs is human nasal epithelial cells (HNECs), which are increasingly being used as a model for the lower airways. HNECs can be obtained via nasal curettage, a nonsurgical and well-tolerated method (16). A direct comparison of paired HBEC and HNEC samples demonstrated that mature airway cell markers and CFTR activity with and without modulator treatment are preserved (15). However, like bronchial brushing, nasal curettage yields a limited supply of cells.

Previous work by our laboratory and others has shown that expression of mouse B cell–specific Moloney murine leukemia virus integration site 1 (Bmi-1) and human telomerase reverse transcriptase (hTERT) enables robust expansion of bronchial epithelial cells that can be differentiated and assayed for CFTR function for up to 15 passages (17, 18). Other groups have applied this method to nasopharyngeal biopsies (19), but, to our knowledge, this technique has not been used for nasal curettage samples. We hypothesized that Bmi-1 and hTERT expression would enhance HNEC growth properties, while maintaining their ability to differentiate into a polarized, pseudostratified epithelium. We propose that this method could be applied to rare-genotype HBECs and HNECs as they become available to create the cellular resources required for personalized medicine and drug discovery.

Here, we created 5 nasal and 3 bronchial Bmi-1/hTERT cell lines that recapitulated primary cell morphology and ion transport function for at least 15 passages. By examining cell lines with *F508del/F508del* and *F508del/S492F* genotypes and comparing them with the parent primary cells, we assessed the fidelity of CFTR modulator responses and the percentage of WT CFTR activity restored. We also compared the in vitro cell culture response to VX-445/VX-661 to the in vivo cell donors’ clinical responses to Trikafta.

We then used this approach to evaluate a potential modulator therapy for patients homozygous for *W1282X*, a nonsense mutation that generates a premature termination codon (PTC) in the *CFTR* transcript, predisposing *CFTR* mRNA to nonsense-mediated decay (NMD) and absent or truncated protein. Without a targetable protein, modulator therapies are ineffective, leaving these patients without treatment options. Recent studies illustrate that the cereblon (CRBN) E3 ubiquitin ligase modulator CC-90009 promotes PTC readthrough by knockdown of a key player in translation termination, the eukaryotic release factor 3a (eRF3a) (20). Using 1 nasal and 2 bronchial *W1282X/W1282X* cell lines, we tested the effects of CC-90009, again showing fidelity between cell lines and primary cells. Not only did we see robust CFTR rescue, but we also observed a dramatic reduction in epithelial sodium channel (ENaC) activity. We then assessed a second eRF3a degrader, SJ6986 (21), and obtained similar results, suggesting that eRF3a degradation represents a generalizable mechanism for the rescue of *CFTR* PTC variants. Finally, we tested CC-90009 and SJ6986 in a previously published but modified *G542X/G542X* bronchial cell line and observed dramatic synergy with the aminoglycoside G418 and robust CFTR functional rescue. We believe the cell lines and data generated here will facilitate the development of treatments for people with CF who currently lack an approved CFTR modulator therapy.

## Results

### Generation of Bmi-1/hTERT nasal and bronchial cell lines and culture optimization.

Primary cell samples were transduced with a lentivirus containing mouse Bmi-1 and hTERT separated by the T2A self-cleaving peptide sequence (Supplemental Figure 1A; supplemental material available online with this article; https://doi.org/10.1172/JCI154571DS1). By these methods, we generated 2 non-CF nasal, 3 CF nasal, and 2 CF bronchial cell lines (Table 1). We confirmed successful vector integration at passage 6 (P6) and P15 by hTERT activity and Bmi-1 protein expression (Supplemental Figure 1, B–F). Conditionally reprogrammed cell (CRC) culture improves HNEC growth capacity (22–24). However, the CRC technique requires coculturing with irradiated or mitomycin-treated NIH3T3 feeder cells. We found that nasal cells could also be effectively expanded in EpiX media (Propagenix) as a feeder-free alternative. A comparison of a representative nasal cell line (UNCNN2T) and its parent cells in CRC and EpiX culture conditions (Supplemental Figure 1G) along with the growth properties of the other cell lines we generated can be found in the Supplemental Figure 2. Cell line differentiation was optimized by comparing UNC air-liquid interface (ALI) media (25), Vertex ALI media (26), and Pneumacult ALI media (STEMCELL Technologies). Pneumacult ALI reproducibly generated a mucociliary epithelial morphology (Supplemental Figure 3) and was used for the remainder of the study except where indicated.

### Bmi-1/hTERT nasal cell lines model primary cell morphology and function.

**Figure 1:**
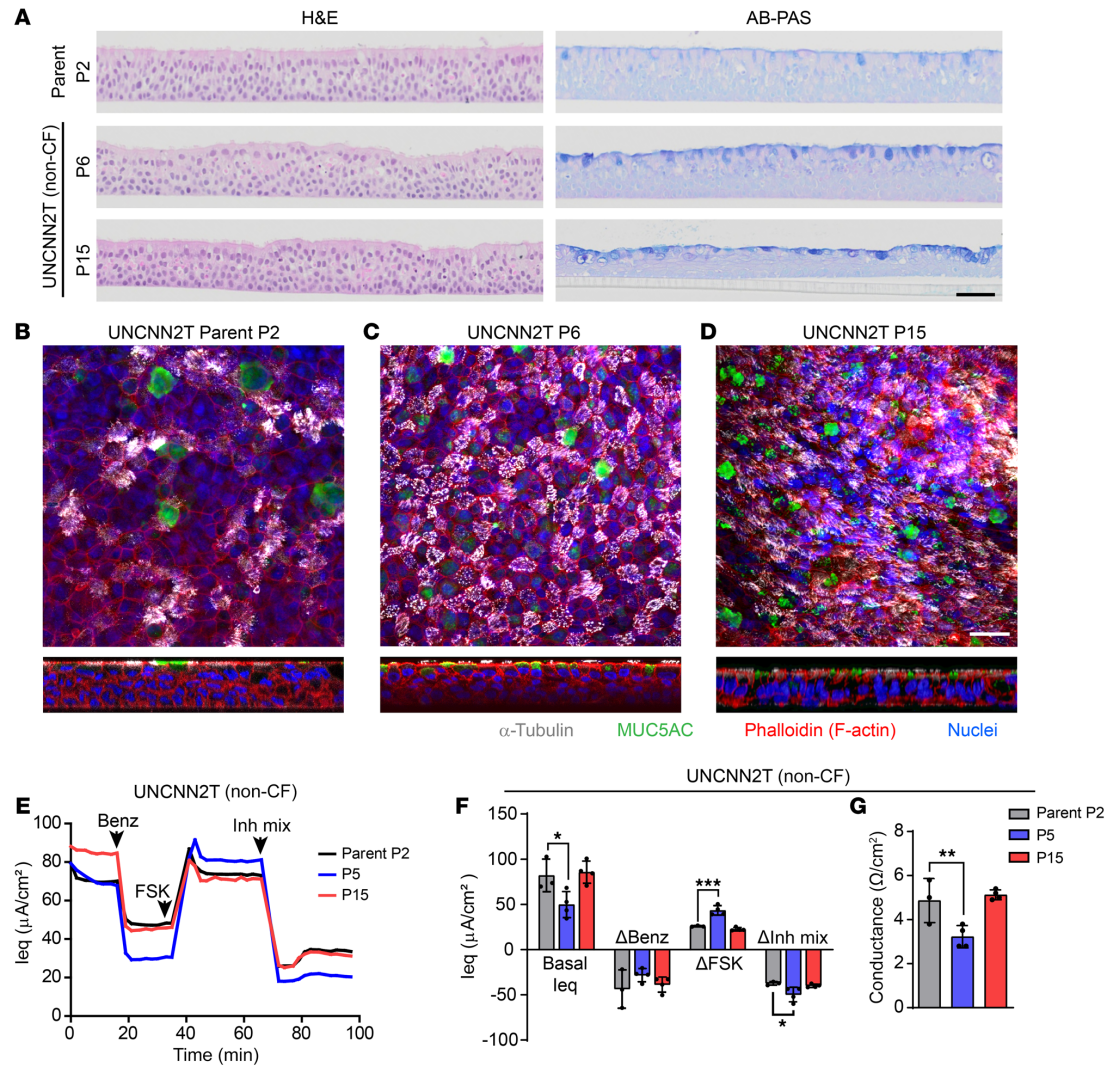
Nasal cell lines model primary cell morphology and ion transport function. (**A**) H&E and AB-PAS staining of UNCNN2T (non-CF) P2 parent cells and cell line at P6 and P15. Scale bar: 50 μm. (**B**–**D**) Whole-mount immunostaining of UNCNN2T P2 parent cells (**B**) and cell line at P6 (**C**) and P15 (**D**). α-Tubulin (white), MUC5AC (green), phalloidin (F-actin, red), and Hoechst (nuclei, blue). Scale bar: 25 μm. (**E**–**G**) TECC-24 measurements of UNCNN2T P2 parent cells and cell line at P5 and P15. (**E**) TECC-24 tracing representing 3–4 replicates. Acute addition of 6 μM benzamil (Benz), 10 μM FSK, and an inhibitor mixture (Inh mix) consisting of CFTRinh-172, GlyH-101, and bumetanide (each at 20 μM), is indicated by arrows. (**F**) Basal Ieq and change in Ieq (ΔIeq) in response to benzamil, FSK, and the inhibitor mixture. *n* = 3–4. (**G**) Baseline conductance values. *n* = 3–4. All data were analyzed using an ordinary linear model and are presented as the mean ± SD. *P < 0.05, **P < 0.01, and ***P < 0.001.

H&E and Alcian blue–periodic acid–Schiff (AB-PAS) staining of a representative non-CF nasal cell line (UNCNN2T) revealed a well-differentiated epithelium at P6 and P15 that was morphologically similar to the parent primary cells at P2 (Figure 1A). These results were confirmed by whole-mount immunostaining, which illustrated the presence of MUC5AC-producing goblet cells and α-tubulin^+^ ciliated cells (Figure 1, B–D). Measurements with a 24-channel transepithelial current clamp amplifier (TECC-24) device demonstrated that parent HNEC electrophysiology was also recapitulated by the UNCNN2T cell line at mid- and late-passage stages but with increased CFTR activity at P5 compared with that of parent cells (Figure 1, E–G). From this, we concluded that Bmi-1/hTERT nasal cell lines model primary HNEC morphology and function for at least 15 passages. Representative histology and whole-mount immunostaining of all other nasal cell lines are shown in Supplemental Figure 4.

### Bmi-1/hTERT nasal cell lines predict the primary cell response to CFTR modulators.

**Figure 2:**
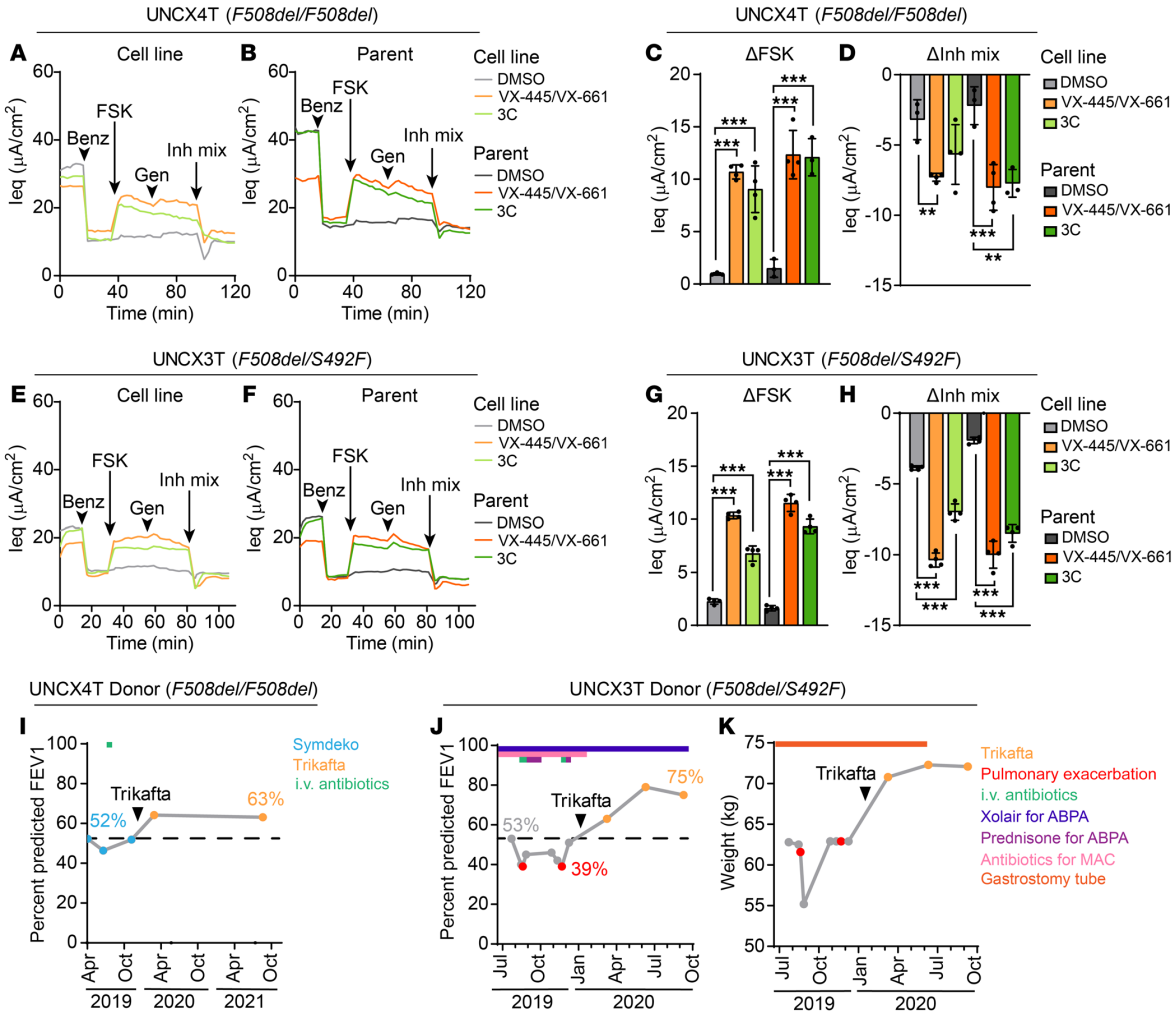
Nasal cell lines predict primary cell and clinical response to CFTR modulators. (**A** and **B**) TECC-24 tracings of the UNCX4T nasal cell line (*F508del/F508del*) (**A**) and parent primary cells (**B**) treated with 0.1% DMSO and VX-445 and VX-661 (each at 5 μM), or a triple corrector combination (3C) containing VX-809/3151/4172 (each at 5 μM). Tracings are representative of 3–4 replicates. Acute addition of the potentiator 10 μM genistein is indicated by arrows. (**C** and **D**) ΔIeq of UNCX4T and parent cells in response to FSK (**C**) and the inhibitor mixture (**D**). *n* = 3–4. (**E** and **F**) TECC-24 tracings of the UNCX3T nasal cell line (*F508del/S492F*) (**E**) and parent primary cells (**F**) pretreated with DMSO, VX-445 and VX-661, or 3C. Tracings are representative of 3–4 replicates. (**G** and **H**) ΔIeq of UNCX3T and parent cells in response to FSK (**G**) and the inhibitor mixture (**H**). *n* = 3–4. (**I**) Change in the percentage of predicted FEV1 before and after Trikafta initiation in the UNCX4T donor (**I**) and the UNCX3T donor (**J**). Blue data points indicate FEV1 measured during SYMDEKO therapy, orange data points indicate FEV1 measured during Trikafta therapy, and red data points indicate FEV1 measured during a CF exacerbation. The treatment course for the UNCX4T and UNCX3T donors is indicated above the respective FEV1 plots and includes the timeline of i.v. antibiotics (green), XOLAIR for ABPA (dark blue), prednisone for ABPA (purple), antibiotics for treatment of *Mycobacterium avium* complex (MAC) (pink). (**K**) Change in weight in kilograms of the UNCX3T cell donor after Trikafta initiation. Gastrostomy tube use and subsequent removal are indicated by an orange bar. All data were analyzed using an ordinary linear model and are presented as the mean ± SD. Post hoc comparisons were performed using the general linear hypothesis test. **P < 0.01 and ***P < 0.001.

The goal of developing patient-derived cell lines is to generate a model in which the primary cell and ultimately the clinical response to CFTR-targeted therapies can be predicted. To test the ability of Bmi-1/hTERT nasal cell lines to predict CFTR modulator responses, we treated our *F508del/F508del* nasal cell line (UNCX4T) with CFTR corrector combinations or a vehicle control (DMSO). Here, and in all subsequent TECC-24 assays, CF cells were pretreated with test compounds and treated acutely with a CFTR potentiator (either genistein or VX-770 as indicated) following CFTR activation with forskolin (FSK). Thus, UNCX4T cells were treated with VX-445 and VX-661, the 2 correctors in Trikafta, or a recently described triple-corrector (3C) combination that combined VX-809 with 2 additional CFTR correctors, 3151 and 4172 (27). UNCX4T cells were then assayed for CFTR function using a TECC-24 device (Figure 2, A, C, and D). VX-445/VX-661 and 3C rescued 23.0% ± 1.4% and 19.5% ± 4.8% of WT CFTR function, respectively (mean ± SD), as determined by dividing the FSK response by the average non-CF nasal cell line response (i.e., 46.5 ± 5.0 μA/cm^2^) (Figure 1F and Supplemental Figure 2H). These data align with preclinical studies of VX-445/VX-661 and clinical observations of Trikafta in *F508del* homozygous populations (28). VX-445/VX-661 and 3C treatment also significantly rescued CFTR ion transport in the parent nasal cells compared with the DMSO control, rescuing 27.3% ± 5.1% and 26.7% ± 3.9% of WT CFTR function, respectively (mean ± SD) (Figure 2, B–D). These data suggest that the 3C combination might be as effective as Trikafta in rescuing *F508del-CFTR* and could represent a therapeutic candidate for those who cannot tolerate or do not respond to Trikafta treatment.

Next, we assessed a *F508del/S492F* compound heterozygous cell line (UNCX3T) for its response to VX-445/VX-661 and 3C (Figure 2, E, G, and H). UNCX3T responded well to VX-445/VX-661 treatment, recapitulating 22.3% ± 0.7% of WT CFTR function (mean ± SD). However, 3C treatment was less effective, producing 14.6% ± 1.5% of WT function (mean ± SD). These findings were mirrored in the UNCX3T parent cells (Figure 2, F–H). Because 3C treatment produced a lower CFTR functional response in UNCX3T, which carries only 1 copy of *F508del*, we posit that this modulator combination does not rescue *S492F-CFTR* as effectively as *F508del-CFTR*. Even so, the response to 3C fell well within the therapeutic window (i.e., 10% of WT CFTR function over baseline) (29) and could serve as an alternative therapy. From these studies, we concluded that Bmi-1/hTERT nasal cell lines generated from CF donors can be used to accurately predict the primary cell response to FDA-approved CFTR modulators and those in preclinical development.

### Nasal cell line functional rescue correlates with the clinical response to Trikafta in 2 patients with CF.

All nasal curettage samples were obtained prior to the 2019 FDA approval of Trikafta. At the time of cell collection, the UNCX4T cell donor (*F508del/F508del*) was prescribed tezacaftor/ivacaftor (trade name SYMDEKO), whereas the UNCX3T cell donor (*F508del/S492F*) was not eligible for CFTR modulators. After nasal cell harvest and cell line generation, both donors became eligible for Trikafta and initiated treatment. Trikafta therapy was highly effective in these individuals, with an 11% and 22% increase in the percentage of predicted forced expired volume in 1 second (FEV1) over a 6-month baseline in the UNCX4T and UNCX3T donors, respectively (Figure 2, I and J). For the UNCX3T donor, Trikafta therapy promoted other significant changes in health, including a reduced frequency of pulmonary exacerbations and need for i.v. antibiotics, cessation of XOLAIR treatment for allergic bronchopulmonary aspergillosis (ABPA), and gastrostomy tube removal following improved weight gain and retention (Figure 2, J and K). Overall, the robust functional response to Trikafta that was observed in the UNCX4T and UNCX3T nasal cell lines in vitro correlated with the cell donors’ positive clinical response to therapy.

### W1282X-CFTR is rescued by the CRBN modulator CC-90009.

Current therapies are not effective at rescuing the truncated protein generated by the *W1282X-CFTR* variant. One proposed treatment strategy is to promote ribosomal readthrough of the PTC to generate full-length protein (30, 31). Low levels of readthrough can be accomplished with high concentrations of aminoglycosides in overexpression cell lines (32). However, clinical readthrough agents are largely ineffective (33, 34), probably because of NMD, a surveillance pathway by which the cell detects and eliminates PTC-containing mRNA transcripts (35). Indeed, a recent study found substantial degradation of the *CFTR* transcript in an individual homozygous for the *W1282X* variant, with the mutated *CFTR* mRNA expressed at only 2.1% of WT levels (36). Thus, many groups have hypothesized that effective treatment of nonsense mutations will also require NMD inhibition (31, 36, 37). Studies in which NMD is bypassed by expressing intron-less cDNA copies of *W1282X-CFTR* have demonstrated that the truncated protein exhibits defective cellular trafficking and gating that can be augmented by CFTR modulators (38, 39). Yet in primary cells, CFTR modulators alone do not rescue function (30). We therefore hypothesized that effective rescue of *W1282X-CFTR* would require a combination of therapeutic approaches to (a) promote PTC readthrough, (b) inhibit NMD, and (c) modulate the resulting CFTR protein.

Recently, a class of CRBN E3 ligase modulators have been shown to significantly improve PTC readthrough by aminoglycoside compounds (20). One of these agents, CC-90009, is currently under investigation for the treatment of relapsed and refractory acute myeloid leukemia (AML) in a phase I clinical trial (NCT02848001). CC-90009 mediates the interaction between CRBN and eRF3a, also known as the G_1_ to S phase transition 1 (GSPT1) protein, which functions as a key player in stop codon recognition and translation termination. Upon interaction with CRBN, eRF3a is ubiquitinated and targeted for proteasomal degradation. Thus, we hypothesized that CC-90009 would further enhance therapeutic readthrough and rescue of *W1282X-CFTR*.

We optimized the CC-90009 dose by treating primary HBECs with escalating concentrations from 0.01 to 10 μM and probing for eRF3a protein knockdown (Supplemental Figure 5A). Knockdown of 85% was achieved with CC-90009 concentrations of 0.1 μM or higher. We then assessed cytotoxicity by measuring lactate dehydrogenase (LDH) release and loss of transepithelial resistance in ALI cultures treated with CC-90009 doses ranging from 0.1 to 1 μM (Supplemental Figure 5, B–D). Cytotoxicity was not observed with 0.1 μM CC-90009, although it was seen at higher doses. Cell morphology was also not altered by 0.1 μM CC-90009 treatment (Supplemental Figure 5E). From this, we concluded that 0.1 μM CC-90009 effectively reduced eRF3a protein expression without causing cytotoxicity or aberrant changes in cell morphology.

**Figure 3:**
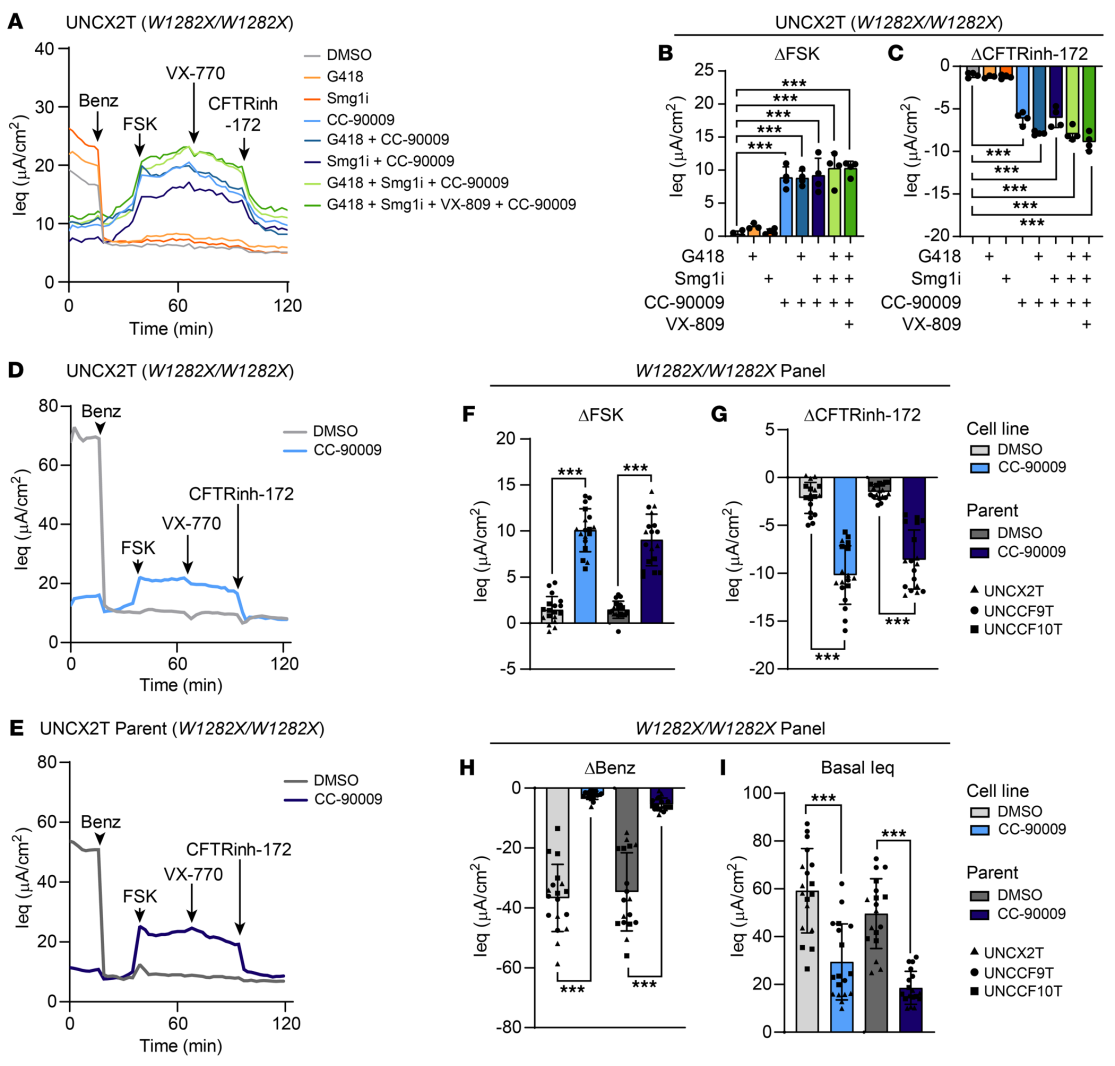
The eRF3a degrader CC-90009 rescues W1282X-CFTR in a panel of cell lines and parent primary cells. (**A**–**C**) TECC-24 measurements of the UNCX2T cell line (*W1282X/W1282X*) treated with 0.1% DMSO, 200 μM G418, 0.3 μM SMG1i, 0.1 μM CC-90009, and 3 μM VX-809, alone or in combination as indicated. Acute addition of the potentiator 10 μM VX-770 is indicated by an arrow. (**A**) TECC-24 tracing representing 3–4 replicates. (**B** and **C**) ΔIeq in response to FSK (**B**) and CFTRinh-172 (**C**). Data were analyzed using ordinary linear models. *n* = 3–4. (**D**–**I**) TECC-24 measurements of a panel of *W1282X/W1282X* cell lines and parent cells treated with 0.1% DMSO or 0.1 μM CC-90009. (**D** and **E**) TECC-24 tracing of the UNCX2T cell line (**D**) and parent cells (**E**). Tracings are representative of the *W1282X/W1282X* panel containing 3 cell donors with 6 replicates per donor. (**F**–**I**) ΔIeq in response to FSK (**F**) and CFTRinh-172 (**G**), benazmil (**H**), and basal Ieq (**I**). Data were analyzed using a linear mixed-effects model with the donor as a random effect factor. *n* = 6 per donor. Post hoc comparisons were performed using the general linear hypothesis test. All data are presented as the mean ± SD. ***P < 0.001.

Having established a safe dose, we then treated a *W1282X/W1282X* nasal cell line (UNCX2T) with combinations of CC-90009, an inhibitor of NMD (Smg1i), an aminoglycoside (G418), and a CFTR corrector (VX-809) and compared the results with a vehicle control (DMSO) and single drug controls (Figure 3, A–C). CFTR function (i.e., FSK and CFTRinh-172 response) was undetectable in vehicle-treated cells or cells treated with Smg1i or G418 alone. However, all combinations that included CC-90009 resulted in substantial CFTR rescue. Unexpectedly, the CC-90009 single drug control rescued 19.1% ± 3.5% of WT CFTR function, indicating that CC-90009 was primarily responsible for the rescue seen in the tested combinations. Unlike previous reports (20), we did not observe synergy between G418 and CC-90009 to rescue *W1282X-CFTR*. The sufficiency of CC-90009 to rescue CFTR was confirmed in a panel of 3 *W1282X/W1282X* cell lines (UNCX2T, UNCCF9T, and UNCCF10T) and in the parent primary cells (Figure 3, D–G), where CFTR was rescued to 21.7% ± 5.0% and 19.5% ± 6.0% of WT CFTR function, respectively. From this, we concluded that CC-90009 could function as a single agent to rescue *W1282X-CFTR* to approximately 20% of WT function.

### CC-90009 diminishes ENaC function and expression.

**Figure 4:**
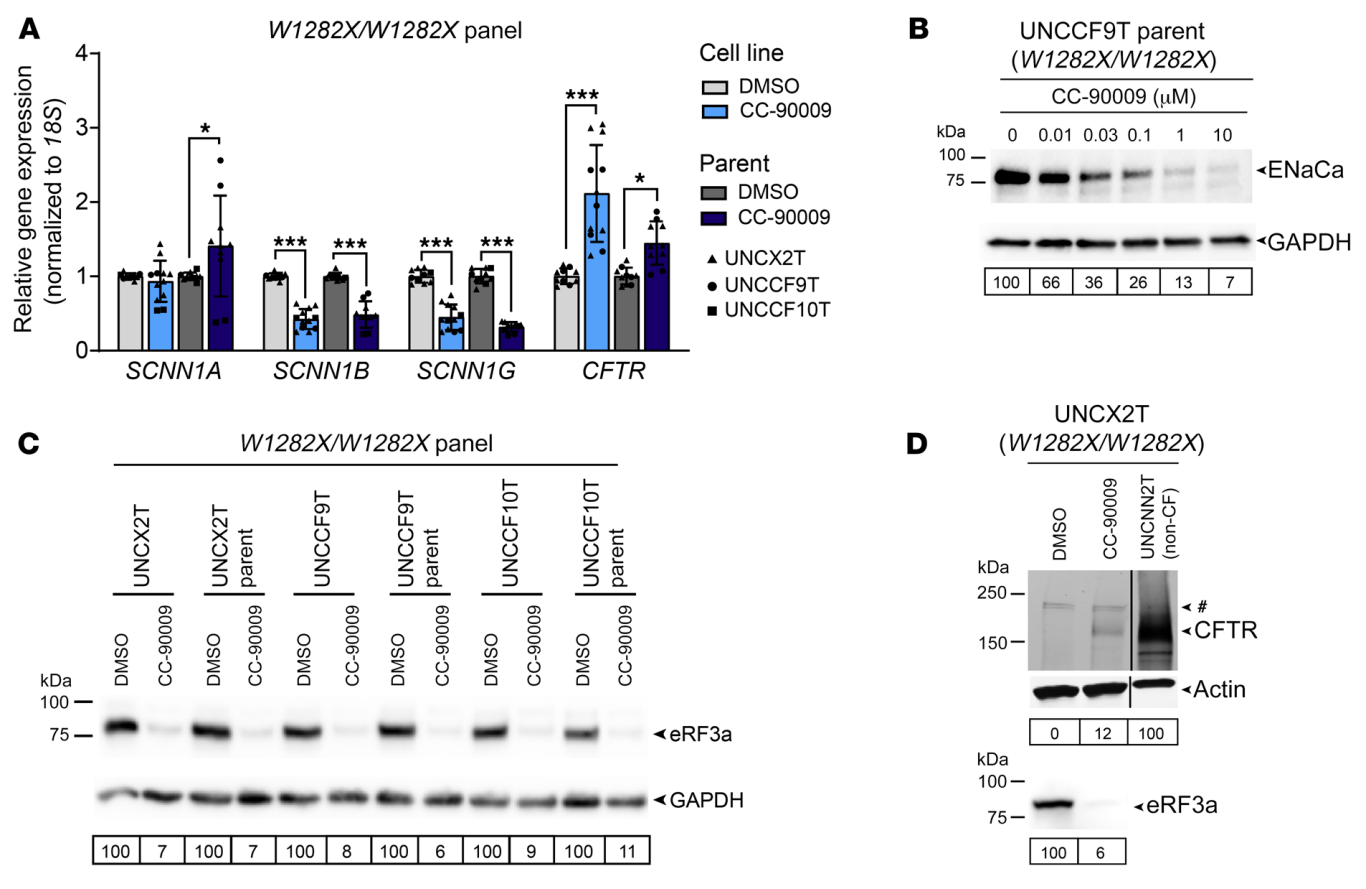
CC-90009 reduces ENaC expression and rescues CFTR by promoting readthrough and full-length CFTR production. (**A**) Relative *SCNN1A*, *SCNN1B*, *SCNN1G*, and *CFTR* mRNA levels by RT-qPCR in a panel of *W1282X/W1282X* cell lines and parent cells. Data were analyzed using a linear mixed-effects model with the donor as a random effect factor and are presented as the mean ± SD. *n* = 2–6 for each cell line. Post hoc comparisons were performed using the general linear hypothesis test. *P < 0.05 and ***P < 0.001. (**B**) Western blot for ENaCa in UNCCF9T parent cells treated with escalating doses of CC-90009. ENaCa expression normalized to GAPDH and relative to the 0.1% DMSO control is quantified below. Cells were grown in Vertex ALI media to increase the level of ENaC expression for detectability by Western blotting. (**C**) Western blot for eRF3a in a panel of *W1282X/W1282X* cell lines and parent cells treated with 0.1% DMSO or 0.1 μM CC-90009. eRF3a expression normalized to GAPDH and relative to the paired DMSO control is quantified below. (**D**) CFTR IP–Western blot (top) of the UNCX2T cell line (*W1282X/W1282X*) treated with 0.1% DMSO or 0.1 μM CC-90009 or the UNCNN2T non-CF cell line. A nonspecific band was observed in all samples and is indicated by a pound sign. CFTR expression normalized to actin and relative to the UNCNN2T non-CF control is quantified below. The same blot was reprobed for eRF3a (bottom). eRF3a expression normalized to actin and relative to the DMSO control is quantified below. All samples were run on the same blot and imaged at the same intensity level.

ENaC plays a vital role in the lungs, balancing CFTR-mediated fluid secretion by regulating sodium absorption across the airway epithelium. Upon treatment with CC-90009, we noted a striking and unexpected decrease in ENaC function, indicated by the response to benzamil, a potent ENaC inhibitor (Figure 3H). Indeed, CC-90009 diminished the benzamil response by 93.4% ± 3.8% and 84.3% ± 6.1% in our panel of *W1282X/W1282X* cell lines and parent cells, respectively. The basal equivalent current (Ieq) was also reduced in the treated cells (Figure 3I), indicating reduced sodium absorption at baseline. To understand this decrease in function, we assessed the mRNA expression of the 3 ENaC subunits a, b, and g (gene names *SCNN1A*, *SCNN1B*, and *SCNN1G*, respectively) (40) (Figure 4A). Treatment with CC-90009 decreased *SCNN1B* and *SCNN1G* mRNA expression in all cell lines and primary cells, while *SCNN1A* mRNA expression was unchanged in the cell lines and increased in the parent cells. By Western blotting, we observed a dose-dependent decrease in ENaCa protein levels to 26% of baseline expression with the 0.1 μM CC-90009 dose (Figure 4B). From this, we concluded that CC-90009 modulates ENaC expression at both the mRNA and protein levels. Even though eRF3a is the only reported target of CC-90009 (41), our data suggest that ENaC or an ENaC regulator may be a previously undescribed target.

### CC-90009 rescues W1282X-CFTR by promoting PTC readthrough.

As expected, CC-90009 promoted eRF3a knockdown in all of the cell lines and primary cells in our *W1282X/W1282X* panel (Figure 4C). eRF3 plays a known role in SURF (SMG1-UPF1-eRF1-eRF3) complex formation, the first step of NMD (42). Previous work by Baradaran-Heravi et al. demonstrated that degradation of eRF3a by CC-90009 leads to partial NMD suppression and an increase in PTC-bearing transcripts (20). We assessed *CFTR* mRNA expression by real-time quantitative PCR (RT-qPCR) and observed a similar increase in expression, indicating that CC-90009 suppressed NMD (Figure 4A). This partial suppression of NMD may explain why we observed no added benefit from treatment with SMG1i (Figure 3, A–C).

eRF3a plays a vital role in PTC recognition. Thus, we hypothesized that the CFTR functional rescue seen in TECC-24 assays would be primarily attributed to PTC readthrough and generation of a full-length CFTR protein. To test this, we immunoprecipitated CFTR followed by Western blotting (i.e., IP-Western blotting) using an antibody targeting a CFTR C-terminal epitope. With this approach, only full-length CFTR would produce a positive signal. We treated a *W1282X/W1282X* nasal cell line (UNCX2T) with and without CC-90009 and compared the IP-Western results with those from a non-CF nasal cell line control (UNCNN2T). Concurrent with eRF3a knockdown, we observed a positive CFTR signal in UNCX2T cells treated with CC-90009 but not in the DMSO control (Figure 4D). This band appeared to have the same molecular weight as CFTR in the non-CF UNCNN2T cell line, further confirming full-length CFTR production. Full-length CFTR abundance was low in UNCX2T cells treated with CC-90009, at approximately 12% of UNCNN2T expression levels. However, work from Boucher and colleagues has shown that even low levels of CFTR restoration can lead to significant functional rescue (43). From this, we concluded that CC-90009 rescued *W1282X-CFTR* through partial suppression of NMD, readthrough of the PTC, and generation of full-length protein.

### An alternate eRF3a degrader, SJ6986, rescues W1282X-CFTR function and inhibits ENaC function.

**Figure 5:**
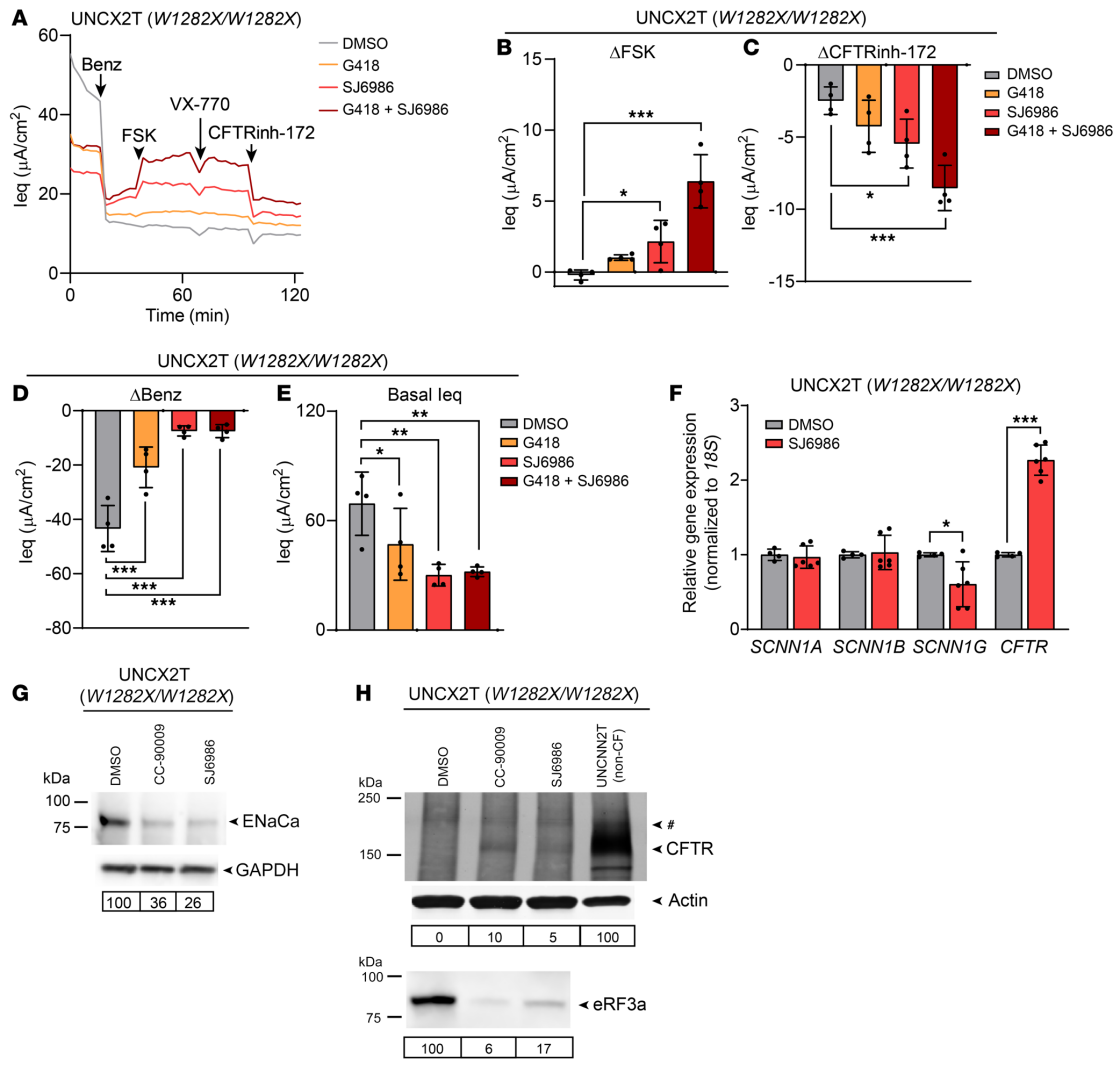
Another eRF3a degrader, SJ6986, rescues CFTR and decreases ENaC expression. (**A**–**E**) TECC-24 measurements of the UNCX2T cell line (*W1282X/W1282X*) treated with 0.1% DMSO, 200 μM G418, and 0.2 μM SJ6986, alone or in combination. (**A**) TECC-24 tracing representing 4 replicates. (**B**–**D**) ΔIeq in response to FSK (**B**), CFTRinh-172 (**C**), and benzamil (**D**). (**E**) Basal Ieq. *n* = 4. (**F**) Relative *SCNN1A*, *SCNN1B*, *SCNN1G*, and *CFTR* mRNA levels by RT-qPCR in the UNCX2T cell line treated with 0.1% DMSO or 0.2 μM SJ6986. *n* = 4–6. (**G**) Western blot for ENaCa in the UNCX2T cell line treated with 0.1% DMSO, 0.1 μM CC-90009, or 0.2 μM SJ6986. ENaCa expression normalized to GAPDH and relative to the DMSO control is quantified below. Cells were grown in Vertex ALI media to increase the level of ENaC expression for detectability by Western blotting. (**H**) CFTR IP–Western blot (top) of the UNCX2T cell line treated with 0.1% DMSO, 0.1 μM CC-90009, or 0.2 μM SJ6986 or the untreated UNCNN2T non-CF cell line. The final lane (UNCNN2T) also appears in Figure 4D. A nonspecific band observed in all samples is indicated by a pound sign. CFTR expression normalized to actin and relative to the UNCNN2T non-CF control is quantified below. The same blot was reprobed for eRF3a (bottom). eRF3a expression normalized to actin and relative to the DMSO control is quantified below. All data were analyzed using ordinary linear models and are presented as the mean ± SD. *P < 0.05, **P < 0.01, and ***P < 0.001.

Nishiguchi et al. recently generated a library of CRBN-modulating chemicals and identified several selective eRF3a degraders (21). One of these degraders, SJ6986, demonstrated higher potency than CC-90009 in their screening assays. Thus, we hypothesized that SJ6986 would also rescue *W1282X-CFTR* by promoting eRF3a knockdown. To establish an optimal dose, we treated a *W1282X/W1282X* nasal cell line (UNCX2T) with increasing concentrations of SJ6986 from 0.2 to 0.8 μM (Supplemental Figure 6). We observed low toxicity and significant CFTR rescue at the 0.2 μM dose. Next, we assessed CFTR function in UNCX2T cells treated with SJ6986 and G418, either alone or in combination, and compared it with a vehicle control (DMSO; Figure 5, A–C). SJ6986 significantly rescued CFTR function but to a lesser extent than was seen with CC-90009, rescuing 4.6% ± 3.2% of WT function. We also observed synergy between SJ6986 and G418, with the combination rescuing 13.7% ± 4.0% of WT CFTR function (Figure 5B). As with CC-90009, we observed a decrease in ENaC function, with an 82.9% ± 4.4% reduction in the benzamil response from baseline (Figure 5D). The basal Ieq was also reduced in SJ6986-treated cells, indicating lower baseline sodium absorption (Figure 5E). At the mRNA level, *SCNN1A* and *SCNN1B* expression remained unchanged. However, *SCNN1G* expression levels were significantly reduced by SJ6986 treatment (Figure 5F). We found that ENaCa protein expression was also substantially decreased by SJ6986 treatment (Figure 5G). From this, we concluded that, like CC-90009, SJ6986 rescued *W1282X-CFTR* function and reduced ENaC expression and function.

We hypothesized that *W1282X-CFTR* functional rescue with SJ6986 was due to PTC readthrough and generation of full-length CFTR protein. To test this, we performed CFTR IP-Western blotting using an antibody targeting a CFTR C-terminal epitope. In the UNCX2T cell line (*W1282X/W1282X*), we observed a positive CFTR signal accompanied by eRF3a knockdown in cells treated with CC-90009 or SJ6986 but not in vehicle-treated cells (Figure 5H). By RT-qPCR, we found that *CFTR* mRNA expression was also increased by SJ6986, indicating partial NMD suppression (Figure 5F). From this, we concluded that SJ6986 rescued *W1282X-CFTR* by the same mechanism as CC-90009 through partial suppression of NMD, readthrough of the PTC, and generation of full-length protein.

### eRF3a degraders synergize with G418 to rescue the G542X-CFTR variant.

Previously, our laboratory published a report on a Bmi-1/hTERT bronchial cell line homozygous for the *G542X-CFTR* variant (UNCCF7T) (44). This cell line was created with separate lentiviruses carrying mouse Bmi-1 and hTERT. In the present study, to boost Bmi-1/hTERT expression, UNCCF7T was transduced with the optimized lentivirus containing both genes (Supplemental Figure 1A). This supplemented cell line was named UNCCF13T to distinguish it from its parent cell line (Table 1).

**Figure 6:**
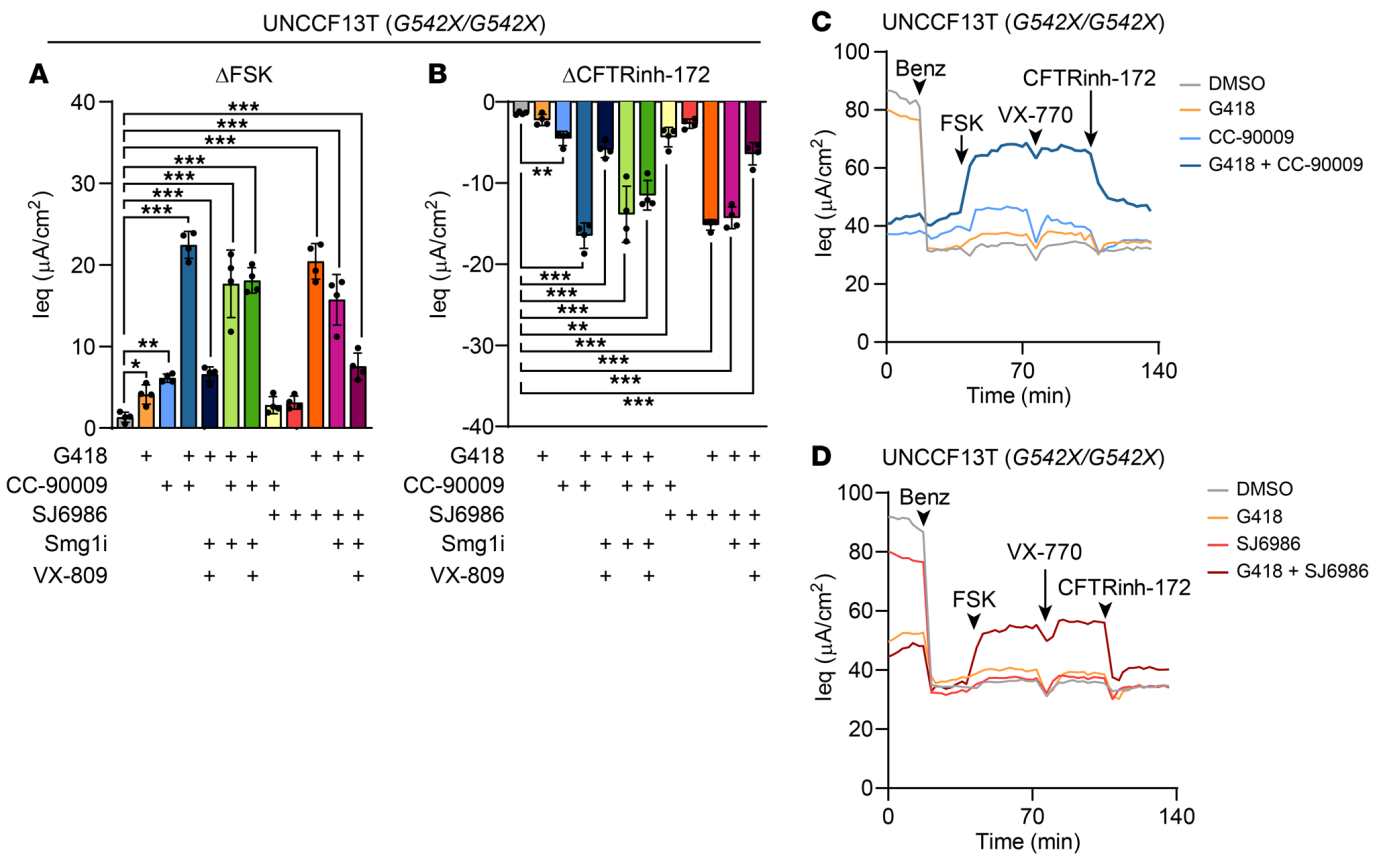
eRF3a degraders synergize with G418 to rescue G542X-CFTR function. (**A**–**D**) TECC-24 measurements of the UNCCF13T cell line (*G542X/G542X*) treated with 0.1% DMSO or combinations of 200 μM G418, 0.1 μM CC-90009, 0.2 μM SJ6986, 0.3 μM Smg1i, and 3 μM VX-809. (**A** and **B**) Change in Ieq in response to FSK (**A**) and CFTRinh-172 (**B**). (**C** and **D**) TECC-24 tracings representing 4 replicates. All data were analyzed using ordinary linear models and are presented as the mean ± SD. *P < 0.05, **P < 0.01, and ***P < 0.001.

To test whether *G542X-CFTR* could be rescued by either CC-90009 or SJ6986, we treated our *G542X/G542X* cell line (UNCCF13T) with CC-90009, SJ6986, G418, Smg1i, and VX-809, either alone or in combination and compared the results with a vehicle control (DMSO) (Figure 6). The CC-90009 and SJ6986 single-drug controls were less effective at rescuing *G542X-CFTR* than rescuing *W1282X-CFTR*, with only marginal functional rescue seen with CC-90009 and no rescue seen with SJ6986. We observed low but statistically significant levels of functional rescue with G418 alone. However, both eRF3a degraders synergized dramatically with G418. The CC-90009/G418 and SJ6986/G418 combinations rescued 48.3% ± 3.6% and 43.9% ± 4.7% of WT CFTR function, respectively. No further improvement was seen with added NMD inhibition (i.e., Smg1i) or CFTR modulation (i.e., VX-809). The discrepancy between our findings with *W1282X-CFTR* and *G542X-CFTR* suggests that sensitivity to and synergy between eRF3a degraders and G418 were highly dependent on mutation context, a phenomenon that has been described previously (45, 46).

## Discussion

The extension of CFTR modulators to 90% of people with CF represents a tremendous victory. However, developing targeted therapies for the remaining 10% will be challenging. This challenge is heightened by the limited availability of rare genotype primary cells. For this reason, the field has turned to cell lines for drug discovery efforts. Preclinical studies rely heavily on Fischer rat thyroid (FRT) cells engineered to express high levels of mutant CFTR (11, 47, 48). While informative, FRT cells do not capture the spectrum of response to CFTR modulators that is routinely observed in clinical trials, even in patient cohorts with the same genotype (6, 7, 49, 50). The inability to mimic patient-specific differences in CFTR regulation and function is an inherent shortcoming of the model (1, 26). Further, because of its robust CFTR overexpression, the FRT model has a greater chance of producing false-positive “hits” (51). This was documented in a study of the *W1282X* variant, in which the modulators VX-809 and VX-770 rescued CFTR function in the FRT model but not in patient-derived nasal epithelial cells (38). Thus, FRT and other overexpression cell lines may not accurately recapitulate mutations that pathologically decrease *CFTR* transcript and protein levels.

Although primary HBECs are the gold standard for CF disease modeling, they can only be expanded for approximately 10 population doublings using conventional growth methods (12). In addition, extensive population doubling often results in squamous differentiation and reduced ion transport (14). Here, we describe the development of Bmi-1/hTERT cell lines derived from primary airway epithelial cells that could be expanded for at least 30 population doublings (15 passages) while recapitulating primary cell morphology and CFTR function. We believe the ability to amply expand and assay CFTR function in patient-derived cells represents a major advantage of this model.

In this study, we directly compared the functional response of patient-derived Bmi-1/hTERT cell lines and the primary cells from which they were developed. Across 5 CF donors (3 nasal and 2 bronchial cell lines carrying a range of CFTR mutations and using both FDA-approved CFTR modulators and those in preclinical development, we found that patient-derived cell lines accurately predicted CFTR rescue in the parent primary cells. In this study, we created 2 nasal cell lines from donors carrying the *F508del* variant to serve as a positive control for an effective modulator response. By testing a Trikafta-like triple combination of VX-445, VX-661, and genistein, we provide proof of concept that CFTR functional rescue in patient-derived cell lines correlated with a positive clinical response to therapy. Although the patient cohort was small, to our knowledge, we are the first group to demonstrate a consensus between patient-derived cell lines, parent primary cells, and patient clinical outcomes. These data highlight the promise of personalized medicine and the predictive power of patient-derived cell lines.

Nonsense mutations in the *CFTR* transcript produce little to no CFTR protein. Thus, therapeutic efforts have largely focused on promoting ribosomal readthrough of the causative PTC. The best-characterized therapeutic agents for inducing readthrough are aminoglycosides (like G418) and ataluren (PTC124). Aminoglycosides have not been approved for clinical use, largely because of high toxicity (52–55). However, a small study demonstrated that short-term intranasal administration of an aminoglycoside partially rescued the CF phenotype of elevated nasal potential difference (56, 57). Ataluren was recently approved by the European Medicines Agency (but not yet by the US FDA) for treatment of Duchenne muscular dystrophy. However, a phase III clinical trial of ataluren in CF was unsuccessful, with no change in the primary or secondary endpoints of FEV1 improvement or the rate of pulmonary exacerbations (34). A recent study demonstrated that aminoglycosides and ataluren promote ribosomal readthrough by distinct mechanisms (58). Aminoglycosides bind directly to the ribosome and increase the probability of near-cognate transfer RNA (tRNA) insertion. By contrast, early evidence indicates that ataluren functions by inhibiting release factor activity (58). Here, we explored an approach to directly inhibit the release factor eRF3a.

During translation, PTCs cause the ribosome to stall and recruit eRF1 and eRF3a. Upon PTC recognition, eRF1 binds the ribosomal A site and, through its interaction with eRF3a, hydrolyzes the peptidyl-tRNA to prompt the release of nascent polypeptides (59, 60). In vitro reporter assays have demonstrated that depletion of either *eRF1* or *eRF3a* by small interfering RNAs (siRNAs) or antisense oligonucleotides (ASOs) promotes PTC readthrough (61, 62). This work was confirmed by Huang et al., who assessed PTC readthrough by knocking down *eRF1* and *eRF3a* with ASOs in a murine hemophilia model (63). Only modest PTC readthrough was observed. However, the group reported striking levels of synergy with aminoglycoside treatment. Another study by Sharma et al. demonstrated PTC readthrough in CFTR following small-molecule eRF1 knockdown (64). This group reported aminoglycoside synergy in FRT and 16HBE gene–edited cell models. In this study, we explored a class of compounds that degrades eRF3a.

Small-molecule eRF3a degraders have been described as potent tumoricidal agents, with activity against AML (41, 65–70). Baradaran-Heravi et al. were the first to assess eRF3a degraders for ribosomal readthrough activity (20). In a panel of human disease models, the authors found that CC-90009 and its analog CC-885 promoted PTC readthrough via eRF3a degradation and that readthrough was further enhanced with the inclusion of aminoglycosides (20). This group also knocked down eRF3a by siRNA and confirmed that the PTC readthrough they observed with CC-90009 was a direct consequence of eRF3a degradation rather than an off-target effect of the compound.

We found that treatment with the eRF3a degraders CC-90009 and SJ6986 substantially rescued CFTR function in patient-derived cell lines carrying the *W1282X-* and *G542X-CFTR* variants. By demonstrating consistent findings between 2 distinct eRF3a degraders, we propose that eRF3a degradation is a generalizable mechanism for CFTR PTC rescue. The robust functional rescue seen in our *G542X/G542X* cell line provided evidence for successful ribosomal readthrough, since CFTR proteins truncated before the 1172 residue are known to have no residual channel function (71). Further, we observed full-length CFTR protein expression by CFTR IP-Western blotting. Assessing the amino acid substitutions at the PTC site in CFTR would be highly informative but is limited by low CFTR protein levels in primary airway epithelial cells. Future studies to understand the amino acid substitutions induced by readthrough agents will require extensive protein purification and/or different model systems.

Genotype-dependent sensitivity to readthrough agents has been described previously (45, 46). Although we did not observe synergy between G418 and CC-90009 to rescue *W1282X-CFTR*, we did observe synergy between G418 and CC-90009 in the *G542X-CFTR* context and between G418 and SJ6986 in both genotypes. We speculate that synergy between eRF3a degraders and aminoglycosides depends on the CFTR genotype, the baseline level of the eRF3a degrader and aminoglycoside responsiveness, and the degree of eRF3a protein degradation by different compounds and doses. Mutation-dependent sensitivity to readthrough compounds further underscores the importance of personalized medicine approaches to assess drug efficacy.

CC-90009 reportedly has little effect on the proteome apart from eRF3a (67, 70). However, Aliouat et al. documented over 2000 genes with significant changes in mRNA expression and protein translation after eRF3a knockdown (72). In the present study, we report an unexpected decrease in ENaC function as a result of CC-90009 or SJ6986 treatment. These findings could have implications for the ongoing clinical evaluation of CC-90009 for the treatment of AML (NCT02848001). Early clinical trial results indicate hypotension as a dose-limiting toxicity and a treatment-emergent adverse event (68). Sodium reabsorption via ENaC is known to determine extracellular fluid volume and regulate blood pressure (73, 74). Thus, we posit that the adverse hypotension observed in CC-90009 clinical trials could be explained by off-target ENaC inhibition. We speculate that ENaC is affected by eRF3a degraders by 1 of 2 possible mechanisms: (a) eRF3a loss directly affects ENaC or an ENaC regulator, or (b) ENaC or an ENaC regulator could share structural similarity with the eRF3a site that binds CC-90009 and SJ6986. Future work will be required to understand the connection between eRF3a degraders and ENaC functional knockdown. Although the effects on ENaC were unexpected, these too could prove therapeutic in the lungs. Increasing airway hydration through ENaC inhibition has been proposed previously as a CF treatment strategy (75).

One concern of therapeutically degrading eRF3a is the possibility of normal termination codon (NTC) readthrough. However, NTCs are more highly regulated than PTCs (63). Further, studies of ataluren-induced readthrough via release factor inhibition demonstrated no adverse effect on translation termination at NTCs (53, 76). While these studies set the precedent that readthrough therapies are safe, future work will be important to determine whether eRF3a degradation affects translation termination at NTCs.

In summary, here we describe a pipeline for developing patient-derived airway epithelial cell lines to model rare CFTR variants. Bmi-1/hTERT nasal and bronchial cell lines are highly representative of primary cell morphology, function, and response to CFTR modulators and correlated with the cell donors’ clinical response to Trikafta therapy. We present the finding that eRF3a degradation by CC-90009 or SJ6986 promoted robust functional rescue of the *CFTR* PTC variants *W1282X* and *G542X*. We observed an off-target but potentially beneficial reduction of ENaC activity following treatment with both eRF3a degraders, and follow-up studies are required to understand the mechanism. Overall, eRF3a degradation may be a promising therapeutic strategy to develop treatments for people with CF carrying PTC variants.

## Methods

### Primary cell isolation and tissue culture.

Primary HNECs were obtained by nasal curettage from 2 non-CF and 3 CF donors, yielding 2.1 × 10^6^ cells on average. Demographics and *CFTR* genotypes are summarized in Table 1. Freshly obtained samples were transferred into lactated Ringer’s solution. Cell dissociation was performed by treatment with DTT (0.5 mg/mL; MilliporeSigma, D0632) and DNase (10 μg/mL; MilliporeSigma, DN25) for 15 minutes. Cells were disaggregated by incubation with Accutase (MilliporeSigma, A6964) for 10 minutes. HBECs from explanted CF transplant lungs were obtained and isolated as previously described (25). HNECs and HBECs were cultured by CRC culturing methods (14, 77) unless otherwise indicated. Briefly, cells were cocultured with irradiated NIH3T3 fibroblasts (provided by Richard Schlegel and Xuefeng Liu at Georgetown University, Washington, DC, who received an original stock from Howard Green at Harvard University, Cambridge, Massachusetts, USA) in the presence of the rho-kinase inhibitor Y-27632. Primary cells were cultured with an antibiotic-antifungal cocktail for the first 2 days.

### Production and titering of the Bmi-1/hTERT lentivirus.

HEK293T cells (ATCC) were thawed and passaged a minimum of 2 times in 100 mm tissue culture dishes (Corning, 430167) with DMEM with 10% FBS and 1% penicillin-streptomycin before transfecting the cells at 85%–95% confluence with 8 μg *pCDH-hTERT-T2A-Bmi-1*, 8 μg *psPAX*, and 4 μg *VSVG* plasmids, using Lipofectamine 2000 (Invitrogen, Thermo Fisher Scientific, 11668-019) according to the manufacturer’s protocol. Cells were maintained at 37°C in 5% CO_2_. Media were changed and collected every 24 hours for up to 72 hours and stored at 4°C. A portion of virus-containing medium was aliquoted and frozen at –80°C, and the remainder was concentrated with the Lenti-X Concentrator kit (Clontech, 631231). Viral titering was performed using 15 μL unconcentrated virus or 1.5 μL concentrated virus with the Lenti-X RT-qPCR Titration Kit (Clontech, 632165). Results were read on an ABI QuantStudio 6 RT-PCR machine.

### Creation of Bmi-1/hTERT growth-enhanced cell lines.

Primary cells were cultured in CRC conditions to P1. At approximately 30% confluence, irradiated NIH3T3 cells were removed with trypsin, and the media were replaced with irradiated NIH3T3-conditioned media (78) containing polybrene (10 μg/mL) and hTERT-T2A-Bmi-1 lentivirus (Supplemental Figure 1A) at an MOI of 2. After 24 hours, cells were washed with 1× PBS and transduced again with fresh virus to increase transduction efficiency. After 48 hours of lentivirus exposure, CRC culture conditions were restored by adding irradiated NIH3T3 cells. Cells were passaged using Accutase at 70%–90% confluence. Growth curves were created by calculating population doublings as the log base 2 of the ending cell number divided by the starting cell number.

### Validation of hTERT expression and activity.

hTERT expression was determined using quantitative analyses of telomerase activity by the combination of the conventional telomeric repeat amplification protocol (TRAP) with RT-qPCR (79, 80). Cells were lysed with a buffer containing 0.5% CHAPS (3-[(3-cholamidopropyl)dimethylammonio]-1-propanesulfonate), 10 mM Tris-HCl, pH 7.5, 1 mM MgCl_2_, 1 mM EGTA, 0.1 mM benzamidine, 10 U/mL RNasin, and 10% glycerol. The protein concentration was measured by detergent-compatible (DC) protein assay (Bio-Rad, 500-0112), and 5 mM β-mercaptoethanol was added to the samples. Each 40 μL reaction contained 2 μg cell lysate diluted in 0.1 mg/mL BSA, 1× TRAP reaction buffer (20 mM Tris-HCl [pH 8.3], 1.5 mM MgCl_2_, 10 mM EGTA, and 50 mM KCl, 50 μM of each deoxynucleotide triphosphate, 910 nM telomerase substrate (TS) forward primer [see sequence below], and 0.4 μg T4 gene protein [New England BioLabs (NEB), M0300S]). The reaction mixture was incubated at 33°C for 5 hours followed by 95°C for 10 minutes to inactivate telomerase. RT-qPCR with iQ SYBR Green Supermix (Bio-Rad, 170-8882) was then performed to quantitate the substrate molecules to which telomeric repeats were added. Each 25 μL reaction contained 300 nM TS forward and reverse primer and 1 μL of the product from the first step. A standard curve was produced by 10-fold serial dilution of HEK293 cell extracts, from 4 μg to 4 ng. All samples were run in triplicate. The TS primer sequences were as follows: 5′-AATCCGTCGAGCAGAGTT-3′ (forward) and 5′-CCCTTACCCTTACCCTTACCCTAA-3′ (reverse).

### Western blot analysis.

Cells were rinsed with cold PBS and lysed in RIPA buffer (Boston Bioproducts, BP-115D) with a Halt protease and phosphatase inhibitor cocktail (Thermo Fisher Scientific, 1861280) for 30 minutes on ice and spun at 16,000*g* for 15 minutes to remove the insoluble fraction. The protein concentration was determined using the DC protein assay (Bio-Rad, 5000112). For each sample, 30 μg protein was loaded onto an SDS-PAGE gel and transferred onto a nitrocellulose membrane. To validate Bmi-1 expression, blots were incubated with anti–Bmi-1 antibody (Cell Signaling Technology, 5856S), followed by goat anti–rabbit IgG (H+L) DyLight800 (Invitrogen, Thermo Fisher Scientific, SA5-10036). Signals were detected and quantified using an Odyssey Image scanner (LI-COR). To determine the effect of CC-90009 on ENaCa and eRF3a expression, blots were incubated with anti-ENaCa (mAb UNC1 19.2.1) (81) and anti-eRF3a antibody (Abcam, ab49878), followed by peroxidase-linked donkey anti–mouse IgG (Thermo Fisher Scientific, 45000679) and donkey anti–rabbit IgG (Thermo Fisher Scientific, 45000682), respectively. Signals were detected using Clarity Western ECL substrate (Bio-Rad, 170-5061), imaged with the Sapphire Biomolecular Imager (Azure Biosystems), and quantified with ImageQuant TL. To normalize protein loading, the blots were reprobed with anti-GAPDH (Proteintech, 60004-1-Ig). Western blot images were cropped around the known molecular weight of the respective proteins. The corresponding complete, unedited blots are included in the supplemental material.

### CFTR IP-Western blotting.

CFTR was immunoprecipitated as described previously (82) in Nonidet P-40 buffer (1% NP-40 Alternative [Calbiochem], 150 mM NaCl, 50 mM Tris, 1 mM EGTA, 4 μg/mL leupeptin, 8 μg/mL aprotinin, 200 μg/mL Pefabloc, 484 μg/mL benzamidine, and 14 μg/mL E64, pH 7.4) using an affinity-purified rabbit polyclonal antibody 155 against amino acids 1465–1480 of human CFTR (83), followed by pulldown with protein A/G Plus-Agarose (Santa Cruz Biotechnology). Samples were separated on 4%–20% gradient SDS-PAGE gels (Bio-Rad) and then transferred onto nitrocellulose. Blots were probed with mouse monoclonal anti-CFTR antibodies 596 and 217 (CFTR Antibody Distribution Program; 1:2000 each) and then with IR Dye 680 goat anti–mouse IgG (Invitrogen, Thermo Fisher Scientific). Anti-actin (Cell Signaling Technology) was used as a loading control. Protein bands were visualized using the Sapphire Biomolecular Imager (Azure Biosystems).

### RNA purification and RT-qPCR.

Total RNA was purified using the Direct-zol RNA Miniprep kit (Zymo Research, R2052) and quantified using a NanoDrop 8000 spectrophotometer (Thermo Fisher Scientific). TaqMan assays for *CFTR* (Integrated DNA Technologies, Hs.PT.58.28207352), *SCNN1A* (Hs01013028_m1), *SCNN1B* (Hs00165722_m1), and *SCNN1G* (Hs00168918_m1) were performed with the Luna Universal Probe One-Step RT-qPCR Kit (NEB, E3006L). Expression of each gene was normalized to *18S* (Hs99999901_s1). All TaqMan probes were purchased from Applied Biosystems except the *CFTR* probe. RT-qPCR was run on Applied Biosystem’s QuantStudio 6 (Thermo Fisher Scientific).

### Mycoplasma testing, DNA fingerprinting, and CFTR genotyping.

Mycoplasma contamination was evaluated by the MycoAlert Plus Mycoplasma Detection Kit (Lonza, LT07-710) with the MycoAlert Assay Control Set (Lonza, LT07-518) according to the manufacturer’s instructions. Mycoplasma-specific enzymes were undetectable in Bmi-1/hTERT cells between P6 and P7 or in the parent primary cells at P2. Luminometry results were read on a FLUOstar Omega microplate reader (BMG LABTECH). Cell line authentication was performed by Labcorp’s Cell Line Testing Division on July 13, 2021, using the PowerPlex 16HS (Promega) for short tandem repeat (STR) DNA profiling. Each cell line was unique among human cell lines and samples present in Cellosaurus (version 1.4.4). Further, the STR profile of each cell line matched that of the parent cells. *CFTR* genotyping was performed by clinically approved laboratories and retrieved from the clinical record.

### Culture at an ALI.

Cell culture inserts were coated with human placenta type IV collagen (MilliporeSigma, C7521). Cells expanded by the CRC culture method were seeded at 2.5 × 10^5^ cells on 12 mm Snapwells (Corning Costar, 3407) or at 3.3 × 10^4^ cells on 6.5 mm Transwells (Corning Costar, 3470) or HTS Transwell 24-well Permeable Support (Corning Costar, 3378) and transitioned to ALI when cells reached confluence. Cells were maintained in Pneumacult ALI medium (STEMCELL Technologies, no. 05001) for 21–32 days after seeding. For drug treatment, compounds were added to basolateral media, and 10 μL or 50 μL was added apically for 6.5 mm and 12 mm Transwells, respectively.

### Whole-mount immunostaining and imaging.

ALI cultures were fixed for 15 minutes on days 21–32 in 4% paraformaldehyde and stained for α-tubulin (MilliporeSigma, MAB1864; 3 μg/mL), MUC5AC (Thermo Fisher Scientific, 45M1; 4 μg/mL), F-actin (phalloidin; Invitrogen, Thermo Fisher Scientific, A22287), and DNA (Hoechst 33342; Invitrogen, Thermo Fisher Scientific, H3570) using species-specific secondary antibodies as previously described (84). Cultures were imaged using a Leica TCS SP8 with a ×63 oil objective.

### Electrophysiology measurements.

The transepithelial electrical resistance and the Ieq were recorded using a TECC-24 as previously described (85). To determine the baseline resistance, 8 initial measurements were collected at approximately 2 minute intervals. Changes in Ieq were determined after the addition of benzamil, FSK, a CFTR potentiator (either genistein or VX-770 as indicated), and a CFTR inhibitor 172 (CFTRinh-172) or an inhibitor mixture containing CFTRinh-172, GlyH-101, and bumetanide as indicated. TECC-24 assays were performed on days 21–32 after seeding. Cell cultures were treated with test compounds prior to the TECC-24 assay as indicated in Table 2.

### Statistics.

All data were analyzed using ordinary linear models or linear mixed-effects models when multiple donors were studied, with the donor as a random effect factor. We performed log transformation before statistical testing when the variance differed greatly between groups, as indicated in the figure legends. Analyses were performed with either the *lm* function in base R or the *lmer* function in the R packages *lme4* and *lmerTest*. Post hoc comparisons were performed using the general linear hypothesis test from the *multcomp* R package. A *P* value of less than 0.05 was considered significant.

### Study approval.

Protocols for informed consent to obtain cells via nasal curettage and from explanted CF lungs were approved under the University of North Carolina’s Office of Human Research Ethics/Institutional Review Board studies 98-1015 and 03-1396, respectively.

## Author contributions

REL, CAL, LH, and SHR conceived the project and designed the experiments with the assistance of JWT and NAC. The Bmi-1/hTERT lentiviral vector was prepared by JTM and LCM. Nasal curettage and review of clinical records were performed by MRK and AJK. Cell line generation and growth curve analyses were performed by REL, CAL, and LH. TMM, CAL, SCG, and REL performed whole-mount immunostaining and confocal imaging. Western blots and RT-qPCR was performed by LH and ECBS. CFTR IP-Western blotting were performed by DMC and MG. Electrophysiology experiments were performed by REL, ECBS, and SCG. REL, LH, JWT, and NAC assessed CC-90009’s effects on PTC readthrough. All of the statistical analysis was performed by HD. REL and CAL composed the manuscript and figures. All authors read, edited, and approved the final version of the manuscript.

## Supplementary Material

Supplemental data

## Figures and Tables

**Table 1:**
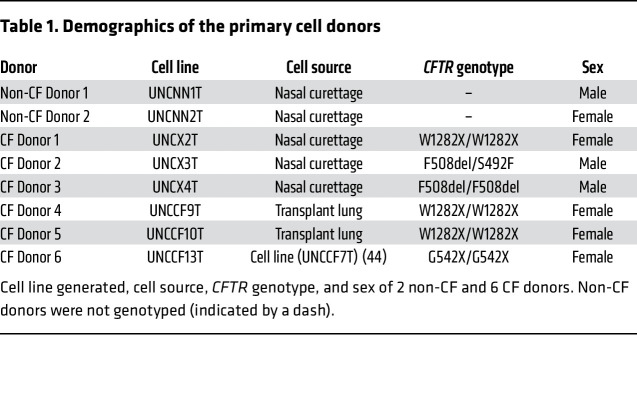
Demographics of the primary cell donors

**Table 2:**
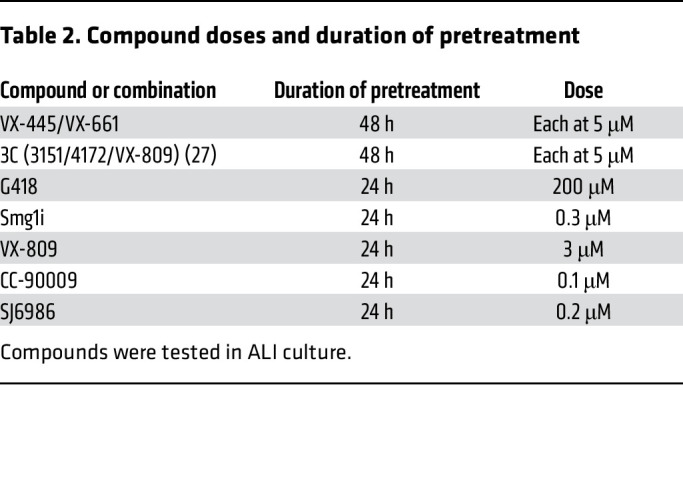
Compound doses and duration of pretreatment

## Version 1 07/28/2022

In-Press Preview

## Version 2 09/15/2022

Electronic publication
