# Supplementary material for: Small-molecule eRF3a degraders rescue CFTR nonsense mutations by promoting premature termination codon readthrough
Source: J Clin Invest. 2022 Sep 15;132(18):e154571. doi: 10.1172/JCI154571 (PMC9479597; doi:10.1172/JCI154571)
Supplement: Supplemental data [file jci-132-154571-s031.pdf]

Supplemental Material

Small molecule eRF3a degraders rescue CFTR nonsense mutations by promoting premature termination codon readthrough

Rhianna E. Lee, Catherine A. Lewis, Lihua He, Emily C. Bulik-Sullivan, Samuel C. Gallant, Teresa M. Mascenik, Hong Dang, Deborah M. Cholon, Martina Gentzsch, Lisa C. Morton, John T. Minges, Jonathan W. Theile, Neil A. Castle, Michael R. Knowles, Adam J. Kimple, Scott H. Randell

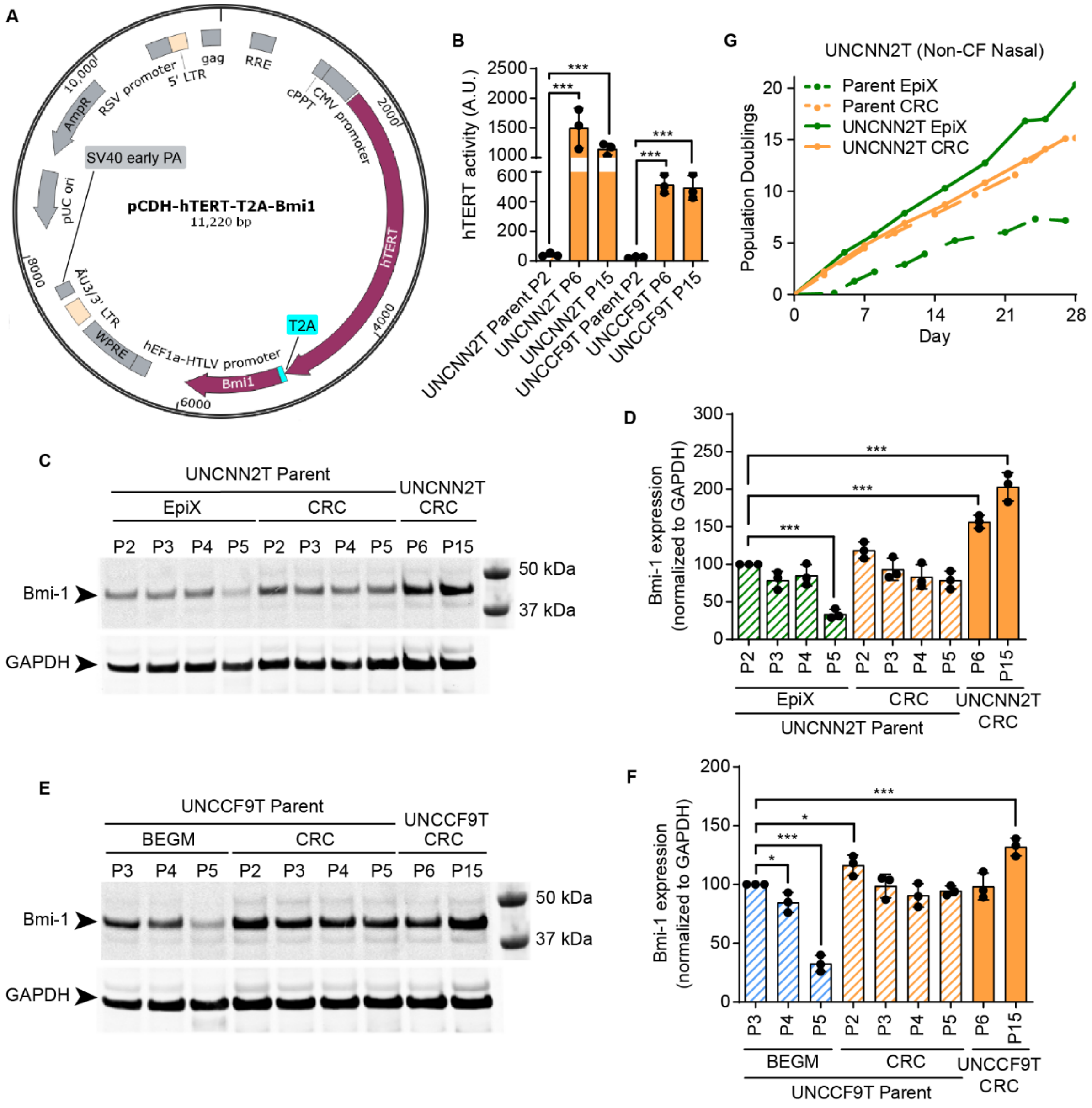

Supplemental Figure 1. Generation of novel Bmi-1/hTERT nasal and bronchial cell lines. A) Vector map of the pCDH lentiviral plasmid containing mouse B cell-specific Moloney murine leukemia virus integration site 1 (Bmi-1) and human telomerase reverse transcriptase (hTERT) sequences linked by the T2A self-cleaving peptide sequence. B) Telomerase activity assay in the UNCNN2T nasal and UNCCF9T bronchial cell lines compared with parent cells. N=3 per condition. Log-transformed data were analyzed using an ordinary linear model with Tukey post-test. C) Western blot for Bmi-1 in the UNCNN2T nasal cell line and parent primary cells. Representative of three independent experiments. D) Bmi-1 normalized to GAPDH and relative to the UNCNN2T parent EpiX P2 sample. N=3. Data were analyzed using an ordinary linear model. E) Western blot for Bmi-1 in the UNCCF9T bronchial cell line and parent primary cells. Representative of three independent experiments. F) Bmi-1 normalized to GAPDH and relative to the UNCCF9T parent BEGM P3 sample. N=3. Data were analyzed by using ordinary linear model. G) Growth curve data for the UNCNN2T cell line and parent primary cells in EpiX media and conditionally reprogrammed cell (CRC) growth conditions. All data presented as mean ± standard deviation (SD). * = p<0.05; *** = p<0.001.

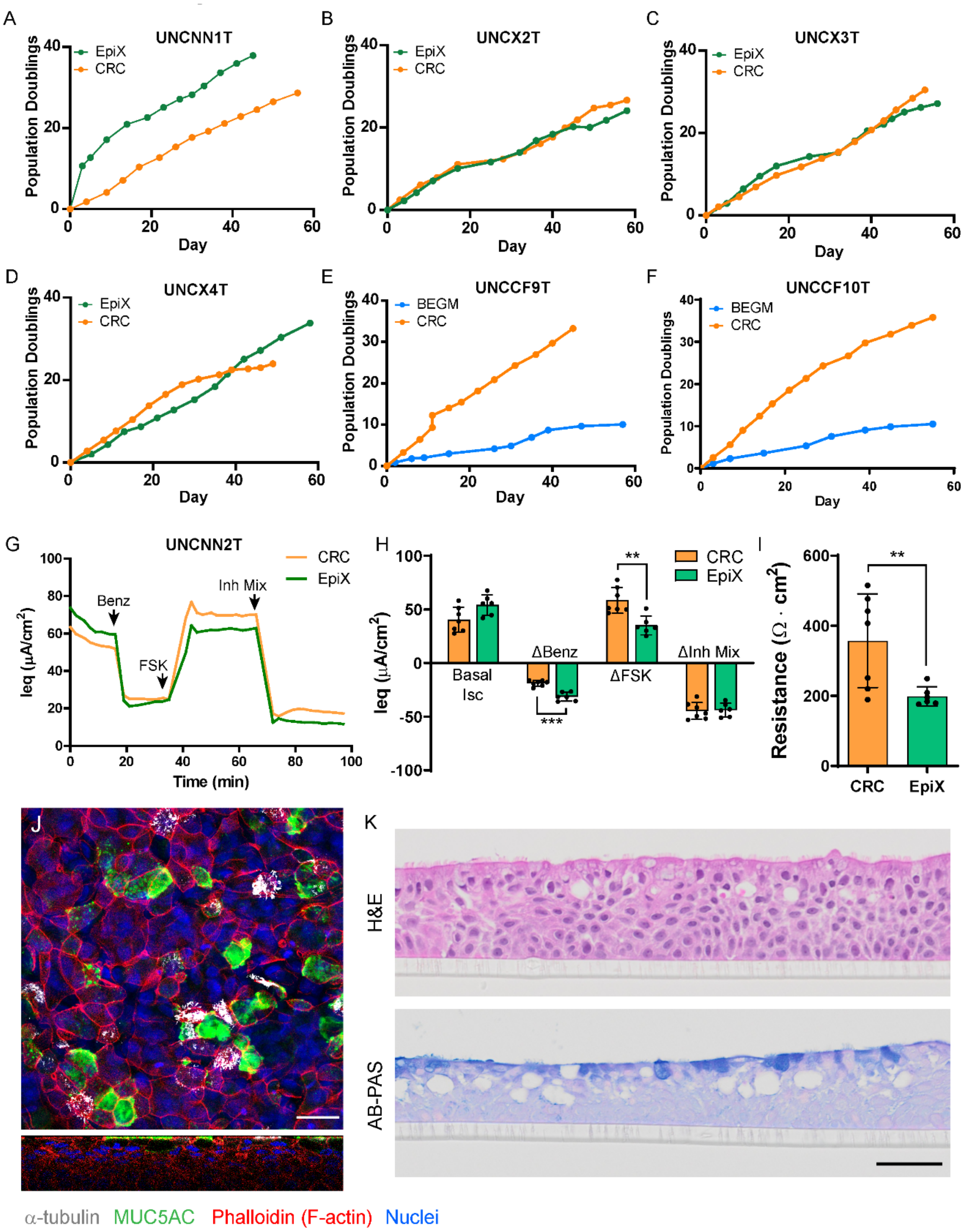

Supplemental Figure 2. CRC and EpiX medium enable robust nasal cell expansion. A-D) Bmi-1/hTERT nasal cell line population doublings over time in CRC and EpiX media: A) UNCNN1T, B) UNCX2T, C) UNCX3T, D) UNCX4T. E-F) Bmi-1/hTERT bronchial cell line population doublings over time in CRC and bronchial epithelial growth media (BEGM): E) UNCCF9T, F) UNCCF10T. G) Representative TECC-24 tracing of UNCNN2T cells expanded by CRC method or in EpiX media and differentiated in Pneumacult ALI media. H) Basal Ieq and ΔIeq in response to Benzamil, FSK, and Inh Mix in UNCNN1T and UNCNN2T cells expanded by CRC method or in EpiX media. N=2-4 for each donor. Data were analyzed using a linear mixed-effects model with the donor as a random effect factor. I) Baseline resistance values. N=2-4 for each donor. Data were analyzed using a linear mixed-effects model with the donor as a random effect factor. J) Whole-mount immunostaining of UNCNN2T P6 cells expanded in EpiX media, α-tubulin (white), MUC5AC (green), Phalloidin (F-actin, red), and Hoechst (Nuclei, blue). Scale bar = 25 μm. K) H&E and AB-PAS staining of UNCNN2T P6 expanded in EpiX. Scale bar = 50 μm. All data presented as mean ± SD. ** = p<0.01; *** = p<0.001.

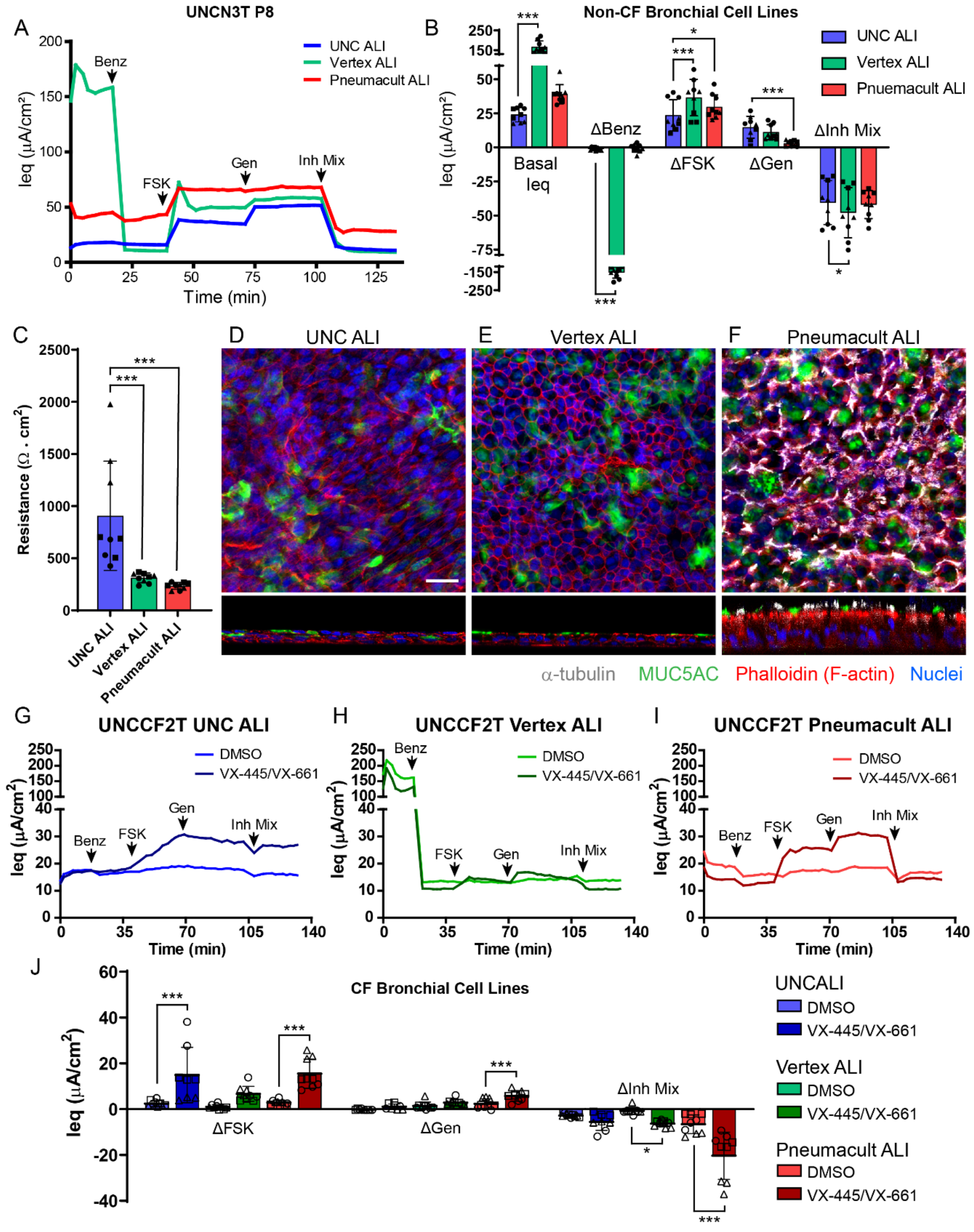

Supplemental Figure 3. Pneumacult ALI promotes differentiation of CF and non-CF Bmi-1/hTERT bronchial cell lines. A-C) TECC-24 measurements of three non-CF Bmi-1/hTERT bronchial cell lines previously published by our lab, UNCN1T P8, UNCN2T P9, and UNCN3T P8, differentiated with UNC ALI, Vertex ALI, or Pneumacult ALI media. A) Representative tracing of UNCN3T P8 cells grown in the three differentiation conditions. B) Basal Ieq and ΔIeq in response to Benz, FSK, Gen, and Inh Mix. Data were analyzed using a linear mixed-effects model with the donor as a random effect factor. C) Baseline resistance values. Log transformed data were analyzed using a linear mixed-effects model with the donor as a random effect factor. B-C) UNCN1T displayed as circles, UNCN2T displayed as squares, and UNCN3T displayed as triangles. N=3 for each cell line. D-F) Whole-mount immunostaining of UNCN3T P8 differentiated with UNC ALI (D), Vertex ALI (E), or Pneumacult ALI (F). α-tubulin (white), MUC5AC (green), Phalloidin (F-actin, red), and Hoechst (Nuclei, blue). Scale bar = 25 µm. G-J) TECC-24 measurements from three CF bronchial cell lines generated from F508del/F508del donors, UNCCF1T P8, UNCCF2T P7, and UNCCF3T P8, pretreated with VX-445/VX-661 (both at 5 µM) or DMSO for

48 hours. G-I) Representative tracings of UNCCF2T grown in UNC ALI (G), Vertex ALI (H), or Pneumacult ALI (I). J) ΔIeq in response to FSK, Gen, and Inh Mix. UNCCF1T displayed as circles, UNCCF2T displayed as squares, and UNCCF3T displayed as triangles. N=2-3 for each cell line. Data were analyzed using a linear mixed-effects model with the donor as a random effect factor. All data presented as mean ± SD. * = $p<0.05$; *** = $p<0.001$.

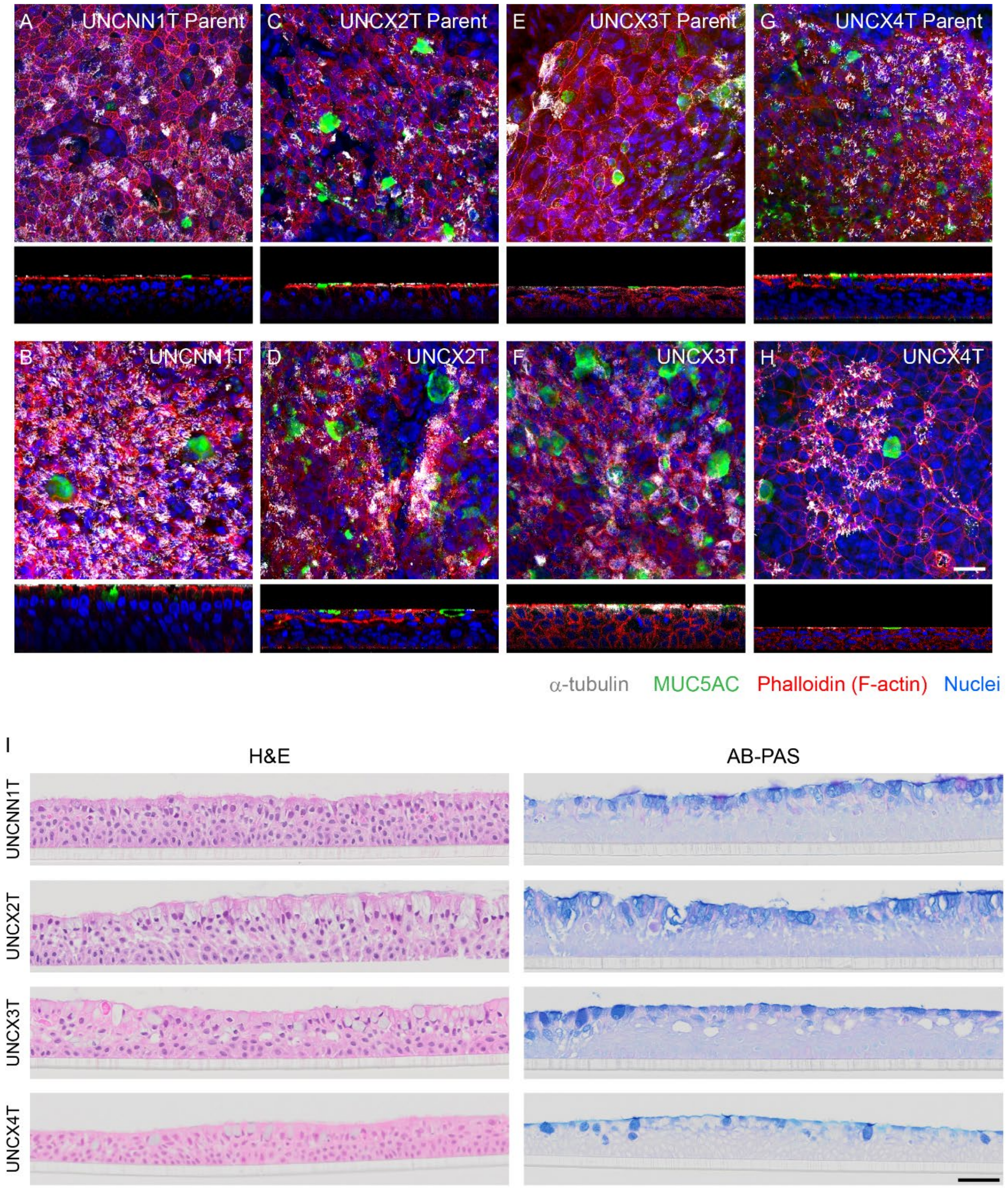

Supplemental Figure 4. Morphology of nasal cell lines. A-H) Whole-mount immunostaining of P2 parent cells and P6 cell line for UNCNN1T (A-B), UNCX2T (C-D), UNCX3T (E-F), and UNCX4T (G-H) cells, α-tubulin (white), MUC5AC (green), Phalloidin (F-actin, red), and Hoechst (Nuclei, blue). Scale bar = 25 μm. I) H&E and AB-PAS staining of UNCNN1T, UNCX2T, UNCX3T, and UNCX4T cells at P5-7. Scale bar = 50 μm.

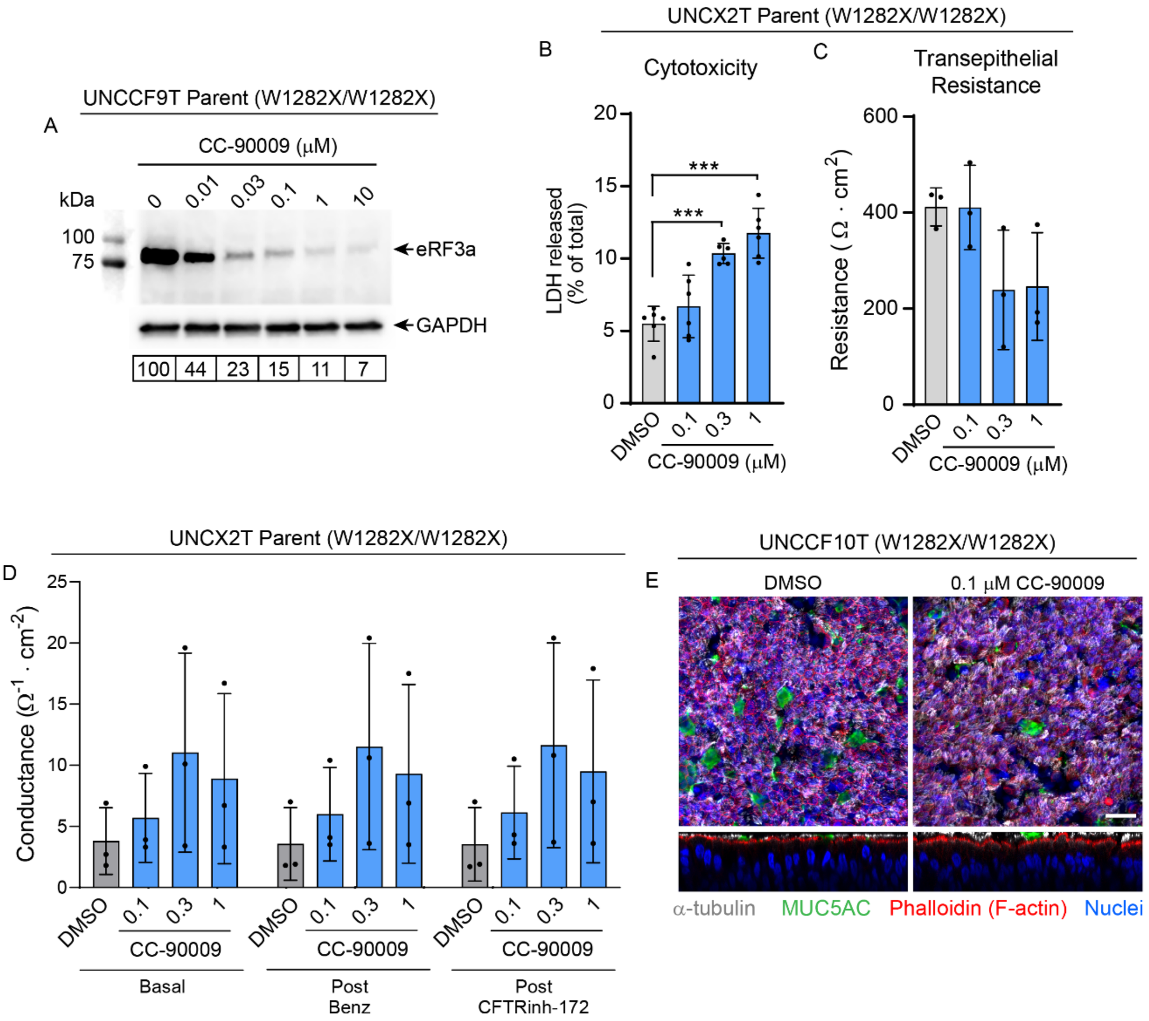

Supplemental Figure 5. CC-90009 dose optimization. A) Western blot for eRF3a in UNCCF9T parent cells pretreated with escalating doses of CC-90009. eRF3a expression normalized to GAPDH and relative to the DMSO control quantified below. B) Cytotoxicity measured by LDH release after treatment of UNCX2T parent cells with escalating doses of CC-90009. N=3. C) Transepithelial resistance of UNCX2T parent cells treated with escalating doses of CC-90009. N=3. D) Conductance of UNCX2T parent cells treated with escalating doses of CC-90009 at baseline (basal), post benzamil, and post CFTRinh-172. N=3. E) Whole-mount immunostaining of UNCCF10T cells pretreated with DMSO or 0.1 μM CC-90009 for 24 hours. α-tubulin (white), MUC5AC (green), Phalloidin (F-actin, red), and Hoechst (Nuclei, blue). Scale bar = 25 μm. All data were analyzed using ordinary linear models and presented as mean ± SD. * = p<0.05; ** = p<0.01; *** = p<0.001.

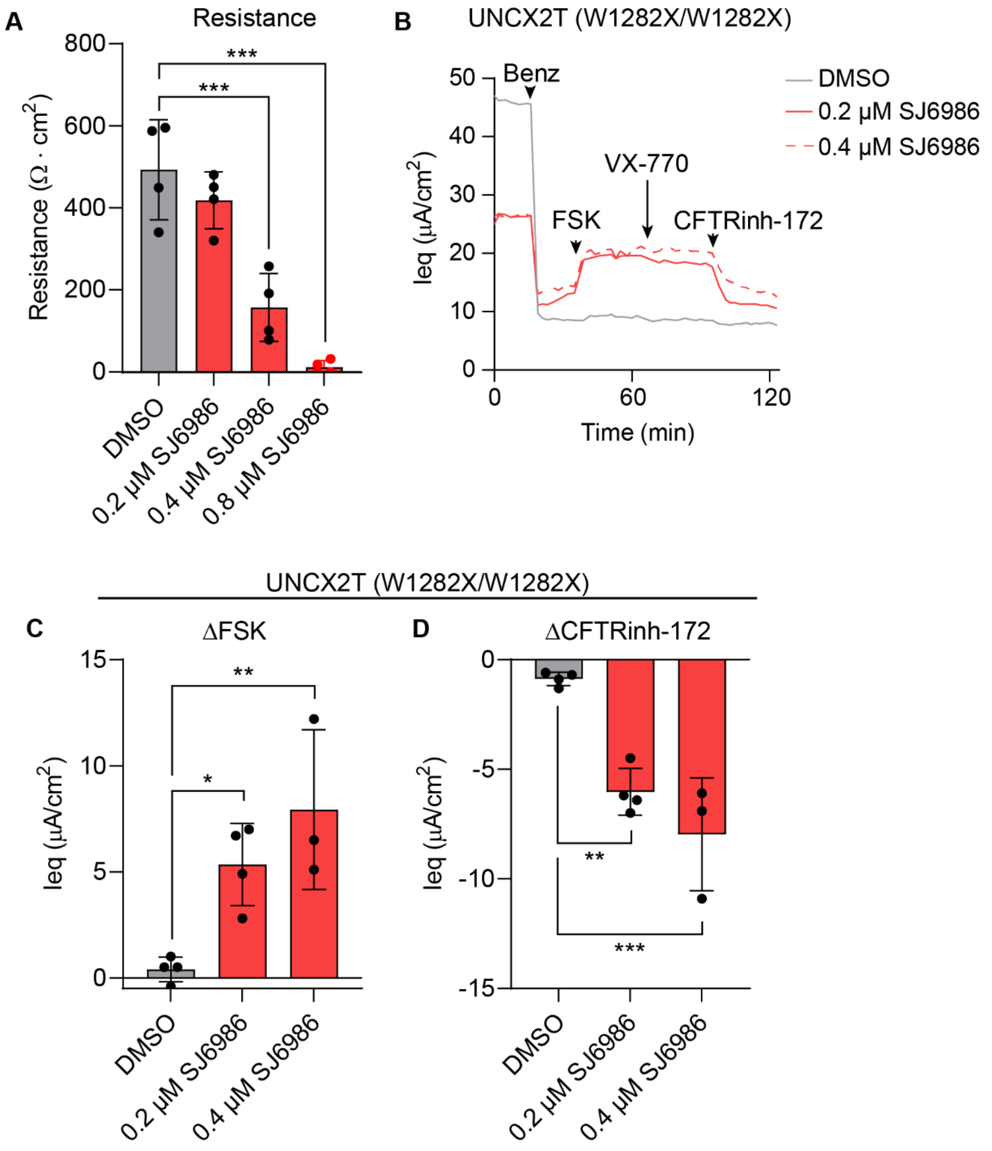

Supplemental Figure 6. SJ6986 dose optimization. A) Transepithelial resistance of the UNCX2T cell line treated with escalating doses of SJ6986. N=4. Red dots indicate cultures with resistances < 100 Ω·cm^2. B-D) TECC-24 measurements of the UNCX2T cell line (W1282X/W1282X) treated with escalating doses of SJ6986. B) TECC-24 tracing representing 4 replicates. C-D) ΔIeq in response to FSK (C) and CFTRinh-172 (D). All data were analyzed using ordinary linear models and presented as mean ± SD. * = p<0.05; ** = p<0.01; *** = p<0.001.

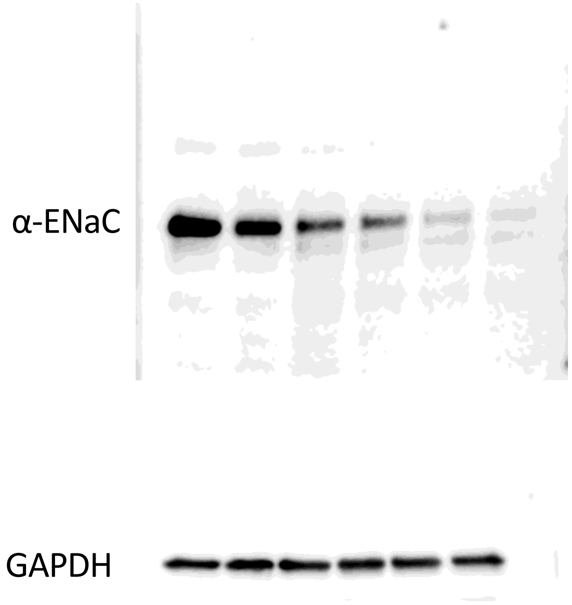

Full blot for Figure 4B

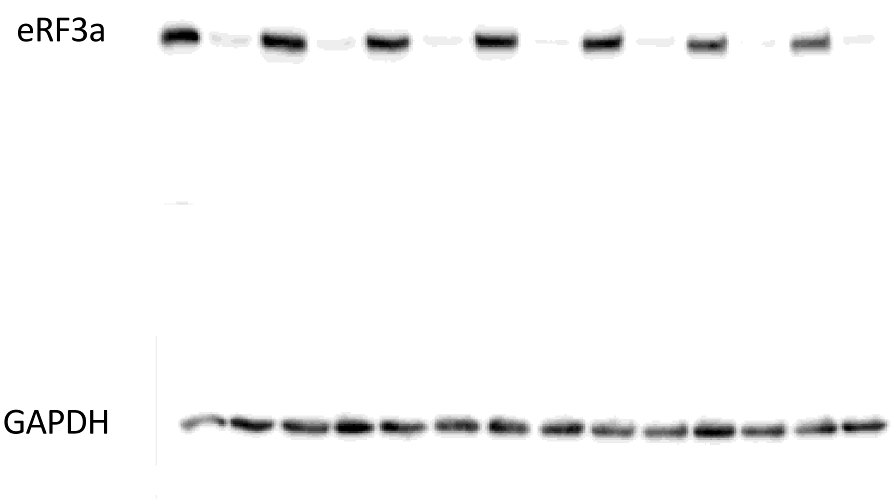

Full blot for Supplemental Figure 4B

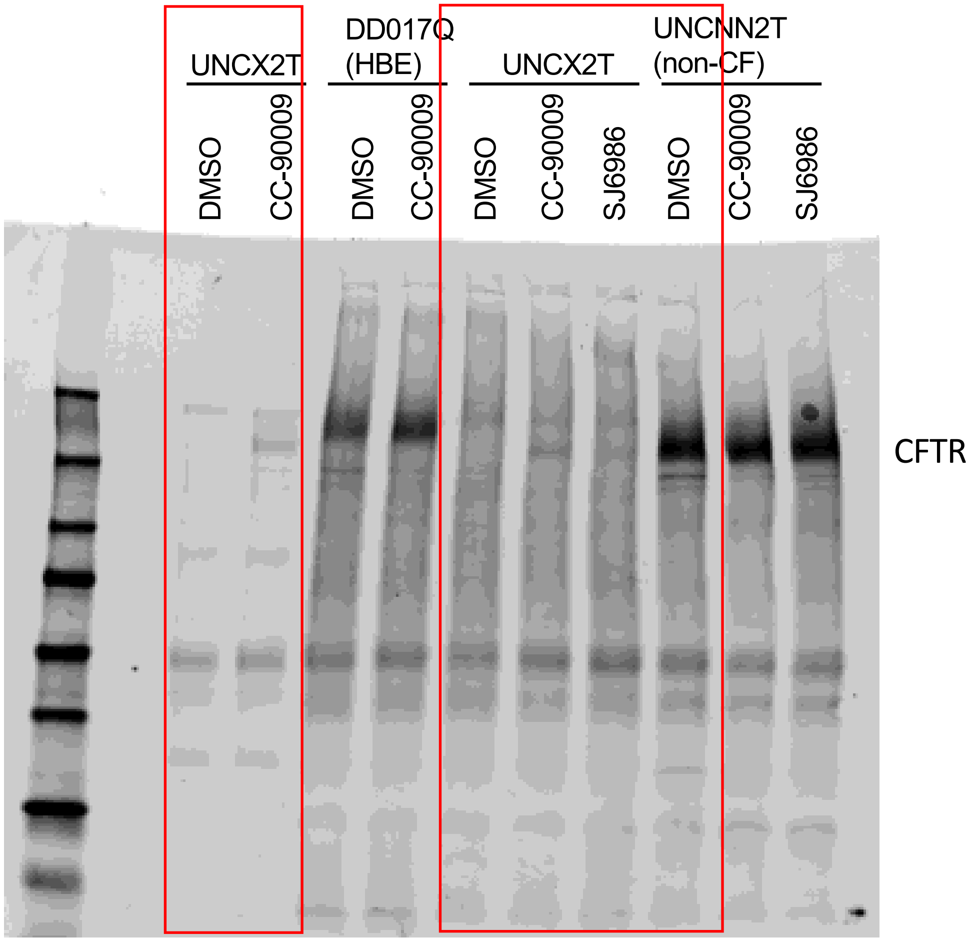

Full blot for Figure 4D and 5H

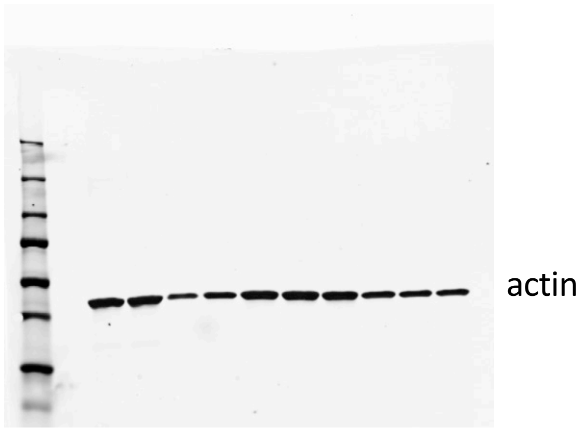

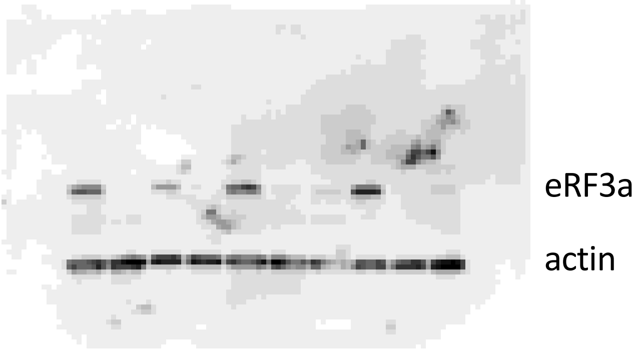

Re-probe actin blot with
anti-eRF3a antibody

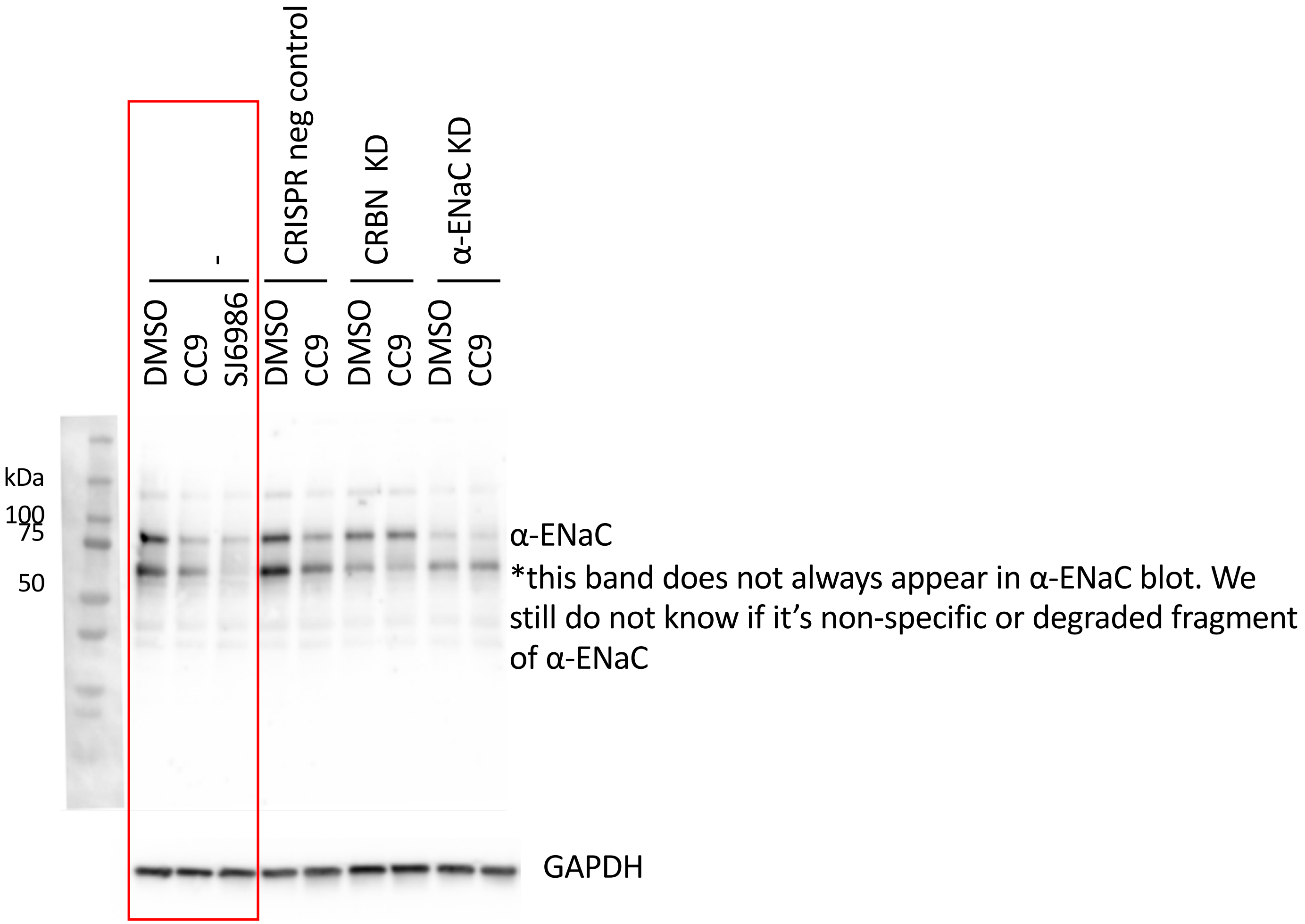

Full blot for Figure 5G

Bmi-1

GAPDH

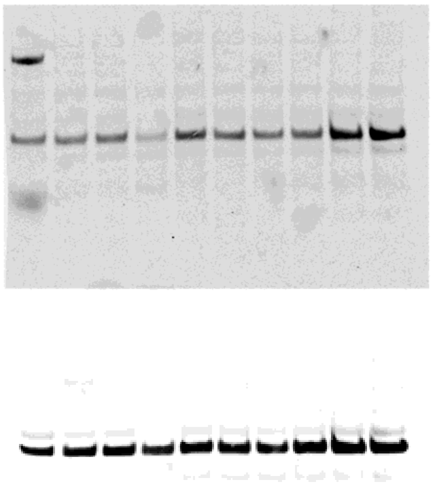

Full blot for Supplemental Figure 1C

Bmi-1

GAPDH

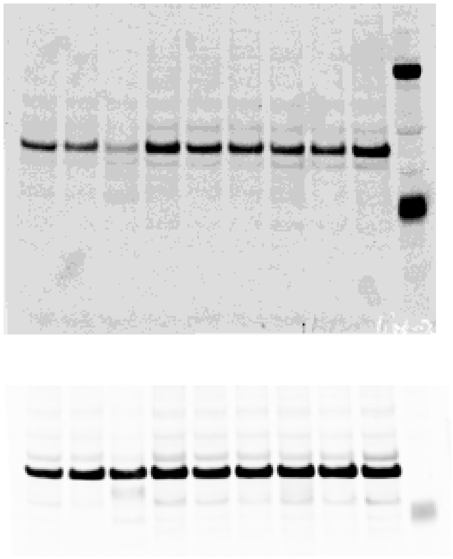

Full blot for Supplemental Figure 1E

eRF3a

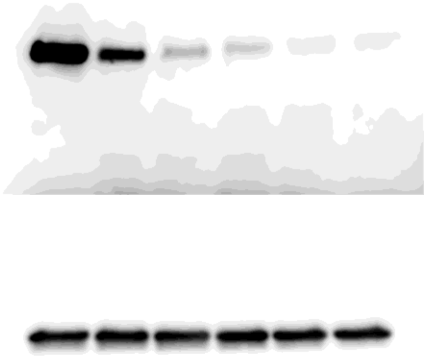

GAPDH

Full blot for Supplemental Figure 5A
